# Ethnoveterinary Knowledge and Biological Evaluation of Plants Used for Mitigating Cattle Diseases: A Critical Insight Into the Trends and Patterns in South Africa

**DOI:** 10.3389/fvets.2021.710884

**Published:** 2021-08-19

**Authors:** Mompati V. Chakale, Mulunda Mwanza, Adeyemi O. Aremu

**Affiliations:** ^1^Indigenous Knowledge Systems Centre, Faculty of Natural and Agricultural Sciences, North-West University, Mmabatho, South Africa; ^2^Food Security and Safety Niche Area, Faculty of Natural and Agricultural Sciences, North-West University, Mmabatho, South Africa; ^3^Centre for Animal Health Studies, Faculty of Natural and Agricultural Sciences, North-West University, Mmabatho, South Africa

**Keywords:** animal health, ethnobotany, food security, livestock, antibacterial, retained placenta

## Abstract

Cattle farming is a traditional agricultural system that contribute to the rural economic, social and cultural values of the communities. Cattle as common with other livestock, are affected by many diseases that cause mortality and economic losses. In many rural households, the use of plants and associated knowledge are popular for managing cattle diseases especially in areas experiencing challenges with conventional veterinary medicine. Evidence on the documentation of indigenous knowledge and biological evaluation of plants used against cattle diseases remain understudied and fragmented. The aim of the review is to collate and analyse the ethnoveterinary knowledge and biological evaluation of plants used against cattle diseases in South Africa. Different scientific databases were systematically explored to extract data from 37 eligible studies. A total of 310 medicinal plants from 81 families used to treat 10 categories of cattle diseases across seven (7) provinces in South Africa. Leguminosae (Fabaceae), Compositae (Astereceae), Asparagaceae, and Xanthorrhoeaceae were the most frequently used plant families. Common plant parts used were leaves and roots. Twenty-seven (27) combination remedies involving 2–6 plants were identified as treatment regimes against cattle diseases. Common preparation methods were infusion and decoction while the administration mode was predominantly unspecified (52%) while oral and topical contributed 26 and 22%, respectively. In terms of diseases, the most treated ones were general systems infection, reproduction disorders and gastrointestinal problems. Currently, an estimated 21% of the 310 plants have been evaluated for diverse biological activities using relevant bioassays related to cattle diseases. Antibacterial activity remained the most studied biological activity. Evidence from the review revealed the significance of ethnoveterinary medicine against cattle diseases especially in rural areas of South Africa. Nevertheless, the use of plants for cattle diseases among other ethnic groups, particularly in the Northern Cape and Western Cape, remain under-studied.

## Introduction

Cattle farming is the backbone of the rural sector and contribute to social and cultural values such as ancestral rituals, *lobola* (bridal) payment, cleansing and sustainable rural livelihoods (1–3). Particularly, cattle are part of livestock farming and a catalyst to enhance household food security and alleviating poverty in small-scale cooperative farming areas. In South Africa, there are about 14 million cattle, which make up 1.6 million dairy cattle (604,781 cows in milk) and 12.5 million beef cattle. Furthermore, ~53 and 47% are in commercial and subsistence systems, respectively (4). However, cattle are often affected by many diseases that cause mortality and economic losses (5–7). Preventing and managing cattle diseases remain a major concern in South Africa as well as in other African countries (8). Therefore, ethnoveterinary medicine (EVM) has become a program that is used to protect and manage animal health and diseases (9–12).

Rural communities often utilize EVM and associated practices to maintain health of wide range of cattle populations (12–14). In South Africa, the use of medicinal plants for treating human diseases have been extensively documented in literature (15–18). However, the neglect relating to ethnoveterinary especially the botanical recording of medicinal plants used to treat animal diseases remain a major concern (19). The need for treatment possibilities is rapidly becoming a key aspect of basic health care within various communities (10, 20). The need to record indigenous knowledge of plants to mitigate their lost due to rapid urbanization and acculturation cannot be over-emphasized (21).

Global interest in EVM practices has increased in the last decade, leading to extensive work especially in Africa (10, 11, 22–24); Asia (25–30); North and South America (31, 32); as well as Europe (33–37). Interest in EVM research is due to readily availability, ease of preparation and administration as well as affordability (13, 38–40). Increasing evidence strongly suggests that EVM has the potential to improve agricultural productivity of local communities (11, 30, 41–43). The current review provides a critical appraisal on the trends and patterns for traditional knowledge and biological evaluation of plants used against cattle diseases in South Africa. It is anticipated that the review will identify existing knowledge gaps and may serve as a reference material for future research efforts in the field of EVM.

## Materials and Methods

### Selection of Scientific Publications

This review was based on the ethnoveterinary studies conducted in South Africa until May 2021. The information on traditional/indigenous knowledge on plants used against cattle diseases in South Africa was extracted from published scientific journals, books, reports from national, and regional, dissertation, theses, conference papers, and reports in South African universities websites/libraries (electronic data repositories), conference proceedings, regulatory and non-governmental organizations. Literature was searched using specific search terms in international online databases such as PubMed, JSTOR, Science Direct, Scopus, and Google Scholar. In the review process, the following search terms were included (singular or plural forms when necessary) in conjunction with South Africa: ethnoveterinary medicine, indigenous knowledge, cattle health care, local cattle husbandry, traditional cattle medicine, animal health anthropology, ethnomedicinal, plant, ethnopharmacology, folk medicine, herbal remedies for cattle diseases, and ethnobotanical papers containing information on plants which was unambiguously linked to a veterinary use. Research articles were also searched by examining bibliographies.

### Selection Criteria

**Figure 1:**
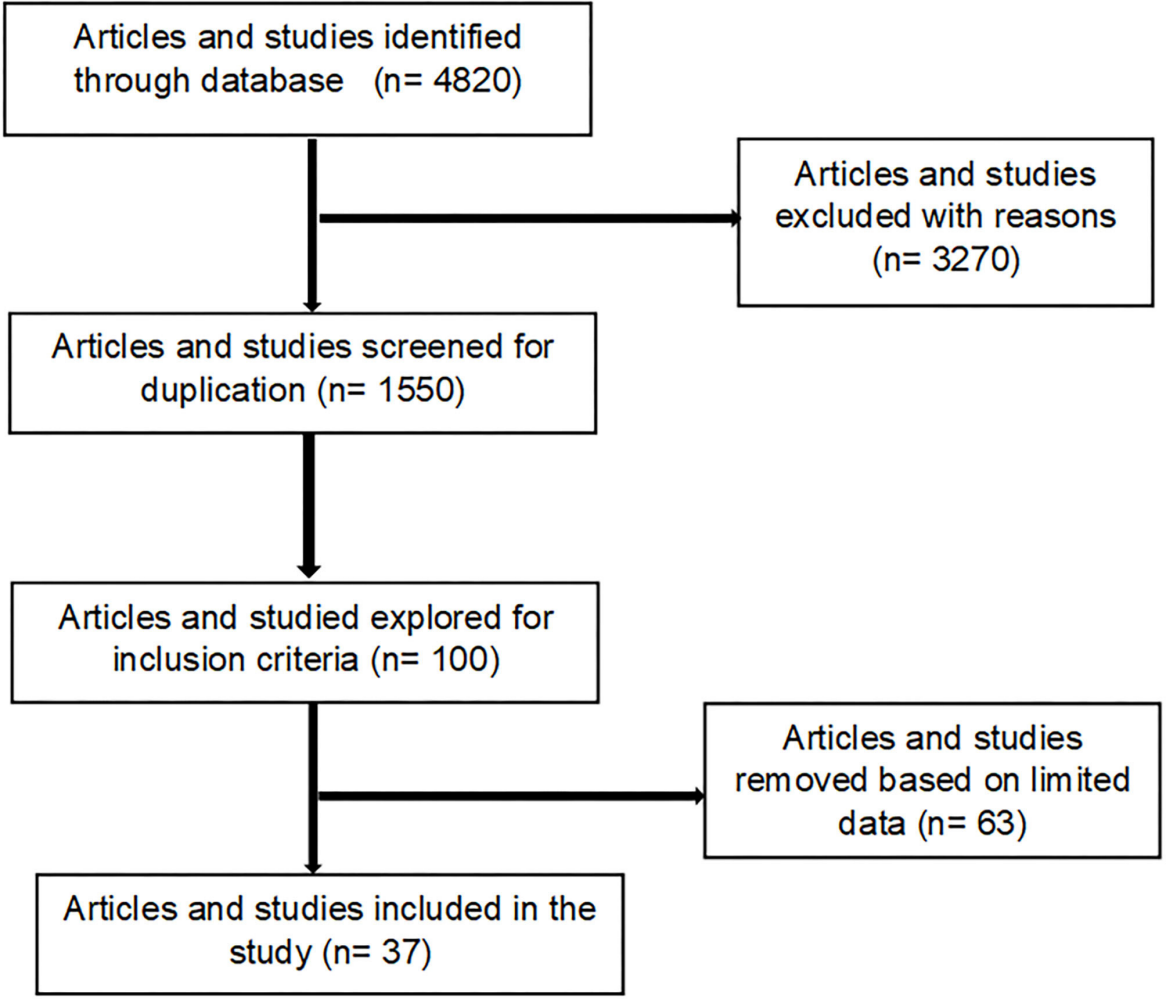
Process used for the selection of articles included in the generated inventory (Supplementary Table S1).

For any article/study to be included in the review, it must include and indicate details of a specific EVM plants relative to its use for treating cattle diseases within the research period (i.e., up to May 2021). For each study, the following information was collected: Latin name of plant used, plant parts, diseases or condition treating, dosage, preparation and mode of administration, the classification of cattle diseases or conditions or therapeutic use of plants. Articles that were excluded were review articles, those solely concerned with modern medicines, or those which cattle were not subject matter. Furthermore, letters, case-reports, manuals, and guidelines, and those reporting only human studies were excluded for this review (Figure 1). The selection of articles was done in four steps. Step one, the relevance of studies was checked based on their title. In the second step, abstracts were evaluated to match to the inclusion criteria. If primary inspection of an abstract of a paper did not give adequate information to make an informed judgment, the full paper was searched in the third step and reviewed by the authors prior to deciding on their inclusion in the review. Finally, those that met the inclusion criteria were retrieved for extra appraisal (Figure 1). All scientific plant names were cross-checked with The Plant List (www.theplantlist.org), while the common names were confirmed using PlantZAfrica (www.pza.sanbi.org).

## Results and Discussion

### South African Ethnoveterinary Medicine Studies Based on Cattle Healthcare

In South Africa, most rural community farmers depend on conventional health practices to preserve and improve their livestock health by preventing and managing diseases (44). Cattle diseases have an influence on the economy and have an impact on cultural practices (45). Ethnoveterinary practices play a greater role in the welfare of cattle as an alternative or an integral part of traditional veterinary practices in rural communities. The use of medicinal plants and indigenous methods/practices for the treatment of diseases is not only limited to humans, but also applies to the treatment of different diseases in cattle (46). Farmers believe that indigenous practices and plants are easy to use/apply, affordable and have less side effects on their livestock. One of the earliest evidence on the use of the EVM was indicated in the work of Gerstner (47). Further studies have been undertaken toward increasing the database of therapies for animal diseases and conditions (Table 1). Studies have been undertaken throughout South Africa with a view to recording indigenous community knowledge of cattle health care, but many rural communities have limited documentation. This justifies the need for the continuation of work in the rest of the country in order to complete the documentation of the EVM used against diseases in cattle.

**Table 1:** An overview of reviewed literature on ethnoveterinary studies on plants used against cattle diseases in South Africa.

**Reference**	**#Province**	**Area/region**	**Ethnic group**	**Number of plant species**	**Number of plant families**	**Diseases/ conditions**	**Voucher specimen deposited**	**Preparation method**	**Administration mode**	**Characteristic of participants**	**Methodological framework (data collection and analysis, techniques)**
Gerstner (47–49)	KZN	Unspecified	Zulu	14	10	9	Unspecified	Unspecified	Unspecified	Unspecified	Ethnobotanical book
Doke and Vilakazi (50)	KZN	Unspecified	Zulu	1	1	1	Unspecified	Unspecified	Unspecified	Unspecified	Ethnobotanical book
Hulme (51)	KZN	Unspecified	Zulu	4	3	4	Unspecified	Yes	Unspecified	Unspecified	Ethnobotanical book
Watt and Breyer-Brandwijk (15)	Southern and Eastern Africa	Unspecified	Unspecified	29	19	10	Unspecified	Yes	Yes	Unspecified	Ethnobotanical book
Bryant (52)	KZN	Unspecified	Zulu	2	1	2	Unspecified	Unspecified	Unspecified	Unspecified	Ethnobotanical book
Pujol (53)	South Africa	Unspecified	Unspecified	2	2	1	Unspecified	Yes	Unspecified	Unspecified	Ethnobotanical book
Roberts (54)	South Africa	Unspecified	Unspecified	3	3	5	Unspecified	Yes	Yes	Unspecified	Ethnobotanical book
Mabogo (55)	LP	Venda	Venda	2	2	3	Unspecified	Yes	Unspecified	Community members	Ethnobotanical book
Hutchings (56)	KZN	Unspecified	Zulu	40	20	20	Unspecified	Yes	Yes	Unspecified	Ethnobotanical book
Masika et al. (57)	EC	Victoria East, Keiskammahoek, Middledrift, Zwelitsha, Mdantsane, Peddie, and Stutterheim	Xhosa	11	10	2	Unspecified	Unspecified	Yes	Farmers	Semi-structure interview, group interview and Rapid Rural Appraisal (RRA)
Masika et al. (58)	EC	Mnqaba-Kulile, Gqumashe, Gwaba, Upper Gxulu, Dongwe, Feni and Fair View, Kubusi, and Kwezana	Xhosa	11	11	7	Yes	Yes	Yes	Farmers, herbalist	In-depth semi-structured interview, convenience sampling, group interview and observation
Dold and Cocks (59)	EC	Ebenezer, Penryn, and Victoria Post	Xhosa	32	26	13	Yes	Yes	Yes	Households, farmers	Questionnaire
Van der Merwe et al. (60)	NW	Madikwe	Tswana	40	21	25	Yes	Unspecified	Unspecified	Farmers, extension officers, traditional healers, knowledgeable elders	RRA, group interview, observation and field walk
Getchell et al. (61)	NW	Seboana, Kromdraai, Vryhof, Setlagole, Kraaipan, and Madibogo	Tswana	4	4	1	Yes	Unspecified	Unspecified	Farmers	Participatory research model
Masika and Afolayan (62)	EC	Unspecified	Xhosa	30	26	8	Yes	Yes	Yes	Farmers and herbalists	RRA, field walk, semi-structure interview guide
Luseba and Van der Merwe (63)	LP	Greater Giyani municipality	Tsonga	15	8	15	Yes	Yes	Yes	Farmers and traditional healers	RRA and interviews
Mahlo (64)	LP	Basani	Unspecified	5	5	4	Unspecified	Yes	Yes	Farmers	Interview, and literature
Moyo (65)	EC	Qolora by-Sea and Nontshinga	Xhosa	3	3	1	Yes	Yes	Yes	Farmers and herbalists	Stratified randomly sampling, interviews
Soyelu and Masika (66)	EC	Amatola Basin	Xhosa	12	10	1	Yes	Yes	Yes	Farmers and community members	structured questionnaires, Snowball sampling
Matlebyane et al. (42)	LP	Ga-Mphahlele, Ga-Dikgale, and Moletjie	Pedi	6	6	7	Yes	Unspecified	Unspecified	Farmers	Semi-structured questionnaires
Luseba and Tshisikhawe (44)	LP	Mutale, Thohoyandou, Nzhelele, and Pundamaria	Venda, Tsonga, and Pedi	26	20	15	Yes	Yes	Yes	Farmers	Open-ended questions, field walks, student's participation in the form of assignments
Beinart and Brown (8)	NW, GP, FS, EC	Mafikeng, Mabeskraal, Garankuwa- Mabopane-Winterveld, QwaQwa, Koppies, Mbotyi—-Mpondoland, Andrew Ainslie, Vimbai Jenjezwa, and Mike Kenyon	Tswana, Sotho, Xhosa, Afrikaners	65	31	24	Unspecified	Yes	Yes	Farmers and community members	interviews
Magwede et al. (67)	LP	Vhembe district	Venda	27	14	2	Yes	Yes	Yes	Farmers, elders and community members	Sem-structured questionnaire, systematic sampling
Kambizi (68)	EC	Pondoland	Xhosa	20	15	10	Unspecified	Unspecified	Unspecified	Herbalists and villagers	Field survey
Mphahlele (69)	LP	Blouberg Municipality	Pedi	5	2	1	Yes	Unspecified	Unspecified	Farmers	Purposeful sampling, Semi-structured interviews
Ramovha and Van Wyk (70)	LP	Vhembe district	Venda	18	9	1	Yes	Yes	Unspecified	Farmers, herders, traditional healers, anthropologists, agriculture extension officers	RRA approach, field surveys, Semi-structured interviews, and observations
Mogale (71)	LP	Tshebela and Ga-Mogano	Pedi	7	7	6	Yes	Yes	Yes	Farmers	Semi-structured interview guide, focus groups discussions, interpretive phenomenological approach
Chitura et al. (72)	LP	Mutale	Venda	9	9	8	Yes	Yes	Yes	Farmers	Purposive sampling, structured questionnaire
Shiba (73)	MP	Chief Albert Luthuli Municipality	Tsonga	7	5	1	Yes	Yes	Yes	Farmers	Questionnaire
Mongalo and Makhafola (74)	LP	Blouberg	Pedi	9	4	Unspecified	Yes	Unspecified	Unspecified	Traditional healers, herbalists	Random sampling, structured questionnaire, s field walks
Mthi et al. (75)	EC	Upper Gqumeya, Ciko, and Goso	Xhosa	6	6	2	Yes	Yes	Yes	Community households	Purposive sampling, semi-structured questionnaire and field observations, analysis
Ndou (76)	NW	Lokaleng, Mogosane, Lokgalong, and Masutlhe	Batswana	24	13	17	Yes	Yes	Yes	Farmers, traditional healers, and community members	Snowball sampling, semi-structured questionnaire, group interviews
Semenya et al. (77)	LP	Ga-Mphahlele	Pedi	30	23	10	Yes	Yes	Yes	Community members	Random sampling, semi-structured questionnaires, field observations
Khunoana et al. (78)	MP	Mnisi/ Bushbuckridge	Tsonga	11	7	7	Yes	Yes	Yes	Farmers, animal health technician, herders, herbalists	Rapid Rural Appraisal, semi-structured interview
Moichwanetse et al. (12)	NW	Dinokana	Batswana	25	18	17	Yes	Yes	Yes	Farmers and herders	Semi-structured interviews, SPSS
Mthi et al. (79)	EC	Upper Gqumeya, Ciko, and Goso	Xhosa	9	8	3	Yes	Yes	Yes	Extension officers, community elders and local authorities	Semi-structured questionnaire, descriptive statistical analysis
Mthi and Rust (80)	EC	Upper Gqumeya, Ciko, and Goso	Xhosa	6	6	1	Yes	Yes	Yes	Community members	Cross-sectional survey using semi-structured questionnaire, purposive sampling

*#Province, EC, Eastern Cape, LP, Limpopo Province, MP, Mpumalanga Province, NW, North West; GP, Gauteng Province; FS, Free State; KZN, KwaZulu-Natal; RRA, Rapid Rural Appraisal*.

Based on inclusion and exclusion criteria, a total of 37 studies on EVM plants used against cattle disease conducted throughout South Africa were identified (Figure 1). In the last 10 years, we observed an increase in publications related to EVM plants used against cattle diseases, indicating an increasing interest in the field. In terms of the geographical distribution of the studies (Table 1), Limpopo province dominated accounting for 32% of the total number of articles. This is due to the province's rich plant diversity and its status as one of the country's hotspots (74). Other major contributions were the Eastern Cape (29.7%), North West and KwaZulu-Natal (13.5%), Mpumalanga (5.4%) while Gauteng and Free State province were the least (2.7%). The most studied ethnic groups were Xhosa (28.9%), baPedi (15.7%), Zulu, VhaVenda, and Batswana (13.2%) and Tsonga (10.5%) while the least responses were from the Basotho and Afrikaner (2.6%). A diverse range of participants involved in the studies were farmers, herbalists, traditional healers, community members (households), knowledge holder (elders), extension officers/animal health technicians, herders, and local authorities (Table 1).

There are numerous methods and approaches used for studying ethnoveterinary knowledge used to treat cattle diseases. Depending on the nature of the knowledge and the degree of certainty researchers had a variety of options. As a result, classification of such a range is critical in order to detect potential systematic patterns in the research literature. Twelve research methodologies were used to collect data, 5 sampling techniques, and 2 analysis methods used in South African EVM studies (Table 1). Semi-structured interview guides were the most commonly used data collection tool as demonstrated in 40% of the reviewed literature while Rapid Rural Appraisal was used in 16% of the articles. It is worth noting that some of the researchers used a variety of methodologies to conduct their research. The majority of studies did not demonstrate the use of approaches and theories to underpin the use of EVM in the treatment of cattle diseases (Table 1). The development of theories and approaches are necessary requirement for the proper development of any field (81). However, the process of developing theories is contentious. Some researchers believe that existing theories should be expanded upon (82) while others believe that new innovative theories should be encouraged in the spirit of plurality (83). Furthermore, none of the articles that took theoretical perspectives proposed a novel EVM theory but were all based on pre-existing theories (76).

### Overview of Medicinal Plants and Families Used in Treating Cattle Diseases

An inventory of plants used against cattle diseases across seven (7) provinces of South Africa was generated (Supplementary Table 1). The plants are arranged in alphabetical order based on the botanical name (with synonyms in the brackets), as well as their families, local names (were available in Setswana/Tswana, Venda, English, Afrikaans, Tsonga, Zulu, and Xhosa), plant parts used, preparation and administration process, and diseases treated are provided. A total of 310 plant species (from 81 families) were used against different cattle diseases. The current review provides a strong indication that South Africa has rich diversity of EVM plants and associated indigenous knowledge. The most frequently mentioned plant which represents 5.5% of the inventory were *Elephantorrhiza elephantina* (Burch.) Skeels, *Aloe ferox* Mill., *Dicerocaryum eriocarpum* (Decne.) Abels, *Senna italica* Mill., *Aloe marlothii* A.Berger, *Boophone disticha* (L.f.) Herb., *Solanum panduriforme* E. Mey, *Spirostachys africana* Sond., *Drimia sanguinea* (Schinz) Jessop, *Pappea capensis* Eckl. & Zeyh, *Calpurnia aurea* (Aiton) Benth., *Gunnera perpensa* L., *Carissa bispinosa* (L.) Desf. ex Brenan, *Clutia pulchella* L., *Gymnanthemum corymbosum* (Thunb.) H.Rob., *Volkameria glabra* (E.Mey.) Mabb. & Y.W.Yuan, and *Ximenia americana* L. Their frequent use and higher number of mentions (3–4 times) in South Africa for diseases in cattle was established in the current review. The relatively high frequency of mentions for these plants is an indication of their effectiveness against diverse diseases in cattle.

**Figure 2:**
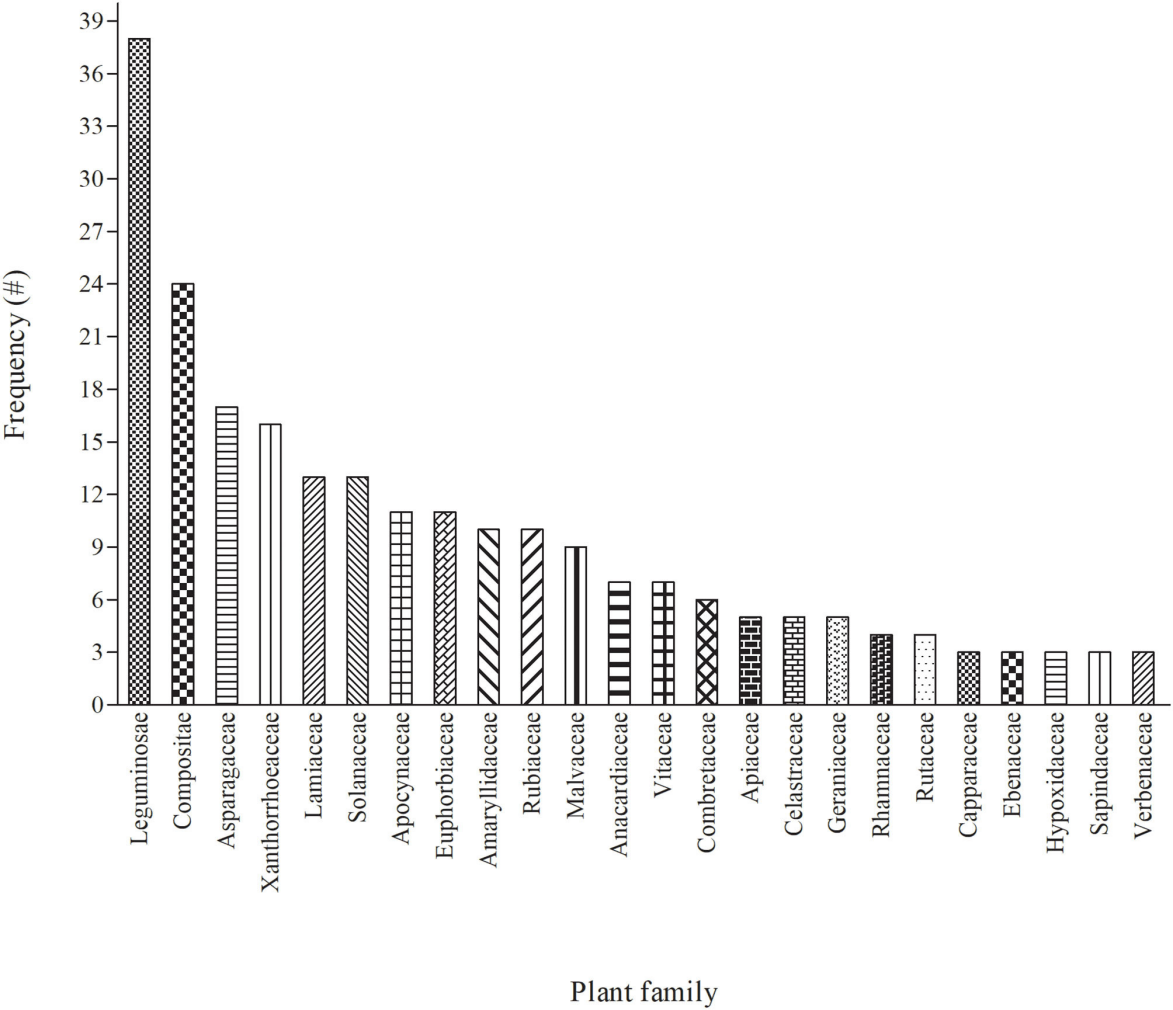
The 24 major (with ≥ 3 mentions) plant families used for treating cattle diseases in South Africa. Each of the remaining 57 plant families were mentioned once or twice each (see Supplementary Tables 1, 2 for details).

In terms of diversity, 81 families were used as herbal medicine to treat and manage cattle diseases in South Africa (Figure 2 and Supplementary Tables 1, 2). Leguminosae/Fabaceae was the most dominant family and contributed 38 plants, followed by Compositae (24), Asparagaceae (17), Xanthorrhoeaceae (16), Lamiaceae and Solanaceae (13), Apocynaceae and Euphorbiaceae (11), Rubiaceae (10), Malvaceae (9) and Vitaceae (7). Leguminosae/Fabaceae had the highest number of plants used to treat cattle diseases which may be attributed to their higher abundance in the study area or due to high bioactivity (84). Similar studies have also been reported from other parts of world where participants mostly use the members of Leguminosae/Fabaceae for the preparation of EVM for the treatment of different livestock diseases (10, 85–87). However, the findings differ from those of other EVM studies in which the other families such as Apiaceae (88), Poaceae (30), Aloaceae (22), Asteraceae (89, 90) and Solanaceae (91) were ranked as the highest. The difference among these studies may be related to the dominant vegetation of the areas or cultural significance (30).

### Plant Parts Used to Treat Cattle Diseases

**Figure 3:**
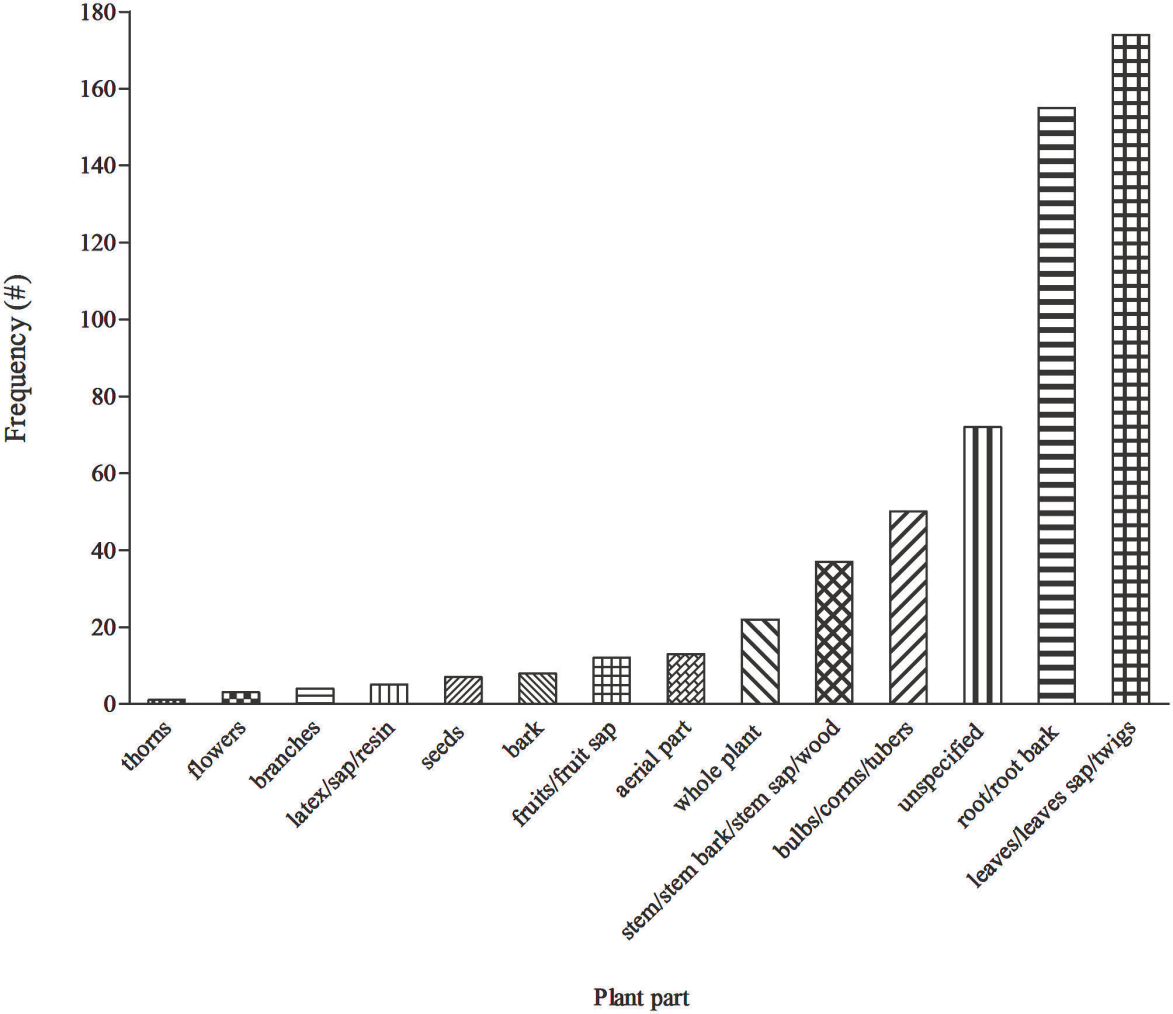
Distribution of medicinal plant parts used to treat cattle diseases in South Africa.

In total, 14 plant parts/components were used for treating cattle diseases in South Africa (Figure 3). Leaves (30.7%) were the most widely used in EVM for treating cattle diseases. The popularity of leaves as one of the most preferred plant part has been a common pattern in South African EVM (46). Preference of leaves over other parts of plants remain common for various reasons including the relatively ease of access when compared to other plant parts. Furthermore, leaves are synthesizing organ for some important plant secondary metabolites that may exert medicinal properties (92–94). From a conservation perspective, individual plants are often not threatened by leaf harvesting for medicinal purpose. Roots constituted 27% and were the second most widely used plant parts, which may be due to rich pool of active compounds, especially terpenes (94). However, the selection of underground parts of the plant including the roots is not viable as it affects plant life and is considered to be highly detrimental to the survival of the whole plant if not done in a sustainable manner (95). As a result, proper harvesting strategies and conservation measures are required to ensure the long-term utilization of medicinal plant resources (95, 96).

### Mono vs. Multi-Plants Application for the Treatment of Cattle Diseases

Even though monotherapy was the most common, the combination of two or more plants were evident in some instances as remedies for treating cattle diseases in South Africa (Table 2). In some instance, a combination of six (6) plants was indicated as treatment remedy for eradicating flea in cattle. Based on the findings by Moichwanetse et al. (12), these type of mixtures are often formulated with more than one plant in order to achieve synergistic or potentiating effects in cattle. Based on the study by Sarswat and Purohit (97), the use of plant mixtures is common for mitigating bovine infertility. Furthermore, the combination of various parts of a plant is commonly used to manufacture medicines for different health conditions in traditional medicine (98–100).

**Table 2:** Examples of plants used in combination therapy for treating cattle diseases in South Africa.

**No of plants**	**Combined plants**	**Plant parts used**	**Preparation and administration methods**	**Disease/condition**	**Reference**
2	*Asparagus setaceus* + *Rhus incisa*	Roots	Infusion	Shock	(59)
2	*Curtisia dentata* + *Rapanea melanophloeos*	Bark	Decoction	Unspecified	(59)
2	*Cussonia spicata* + *Olea europaea*	Bark + leaves	Decoction	Endometritis/ vaginitis	(59)
2	*Dicoma galpinii* + *Senna italica*	Roots	Infusion, oral	Gala	(76)
2	*Grewia flava* + *Ziziphus zeyheriana*	Roots	Decoction, oral	Diarrhea	(76)
2	*Helichrysum caespititium* + *Artemisia afra*	Roots + leaves	Decoction, oral	Coughs	(76)
2	*Hippobromus pauciflorus* + *Protorhus longifolia*	Bark	Decoction	Heartwater, diarrhea	(59)
2	*Pelargonium reniforme* + *Plumbago auriculata*	Roots	Decoction	Diarrhea	(59)
2	*Pelargonium sidoides* + *Ziziphus zeyheriana*	Unspecified	Decoction	Anthelmintics	(15)
2	*Phoenix reclinata* + *Arctotis arctotoides*	Roots + leaves	Decoction, topical	Foot rot	(59)
2	*Ziziphus zeyheriana* + *Helichrysum caespititium*	Roots	Decoction, oral	Pains	(76)
3	*Bulbine abyssinica* + *Solanum lichtensteinii* + *Withania somnifera*	Roots	Infusion, oral	Internal sores	(76)
3	*Drimia sanguinea* + *Senna italica* + *Elephantorrhiza elephantina*	Bulb + roots + bulb	Maceration, oral	Intestinal parasites	(12)
3	*Drimia sanguinea* + *Ziziphus oxyphylla* + *Ziziphus mucronata*	Bulb + roots+ roots	Poultice, topical	Cleaning the kidney	(12)
3	*Hypoxis hemerocallidea* + *Aloe vera* + *Pouzolzia mixta*	Bulb + leaves + roots	Maceration, oral	Heart problems	(12)
3	*Leucas capensis* + *Brachylaena ilicifolia* + *Aloe ferox*	Leaves + sap	Decoction	Unspecified	(59)
3	*Peltophorum africanum* + *Elephantorrhiza elephantina* + *Jatropha zeyheri*	Bulb + roots + bulb	Maceration, oral	Constipation	(12)
3	*Plectranthus laxiflorus* + *Eucomis punctata* + *Kedrostis africana*	Unspecified	Decoction	Gallsickness	(59)
3	*Senna italica* + *Ziziphus zeyheriana* + *Cadaba aphylla*	Roots	Decoction, oral	Pains	(76)
3	*Solanum campylacanthum* + *Helichrysum caespititium* + *Withania somnifera*	Roots	Decoction, oral	Pain	(76)
3	*Solanum lichtensteinii* + *Bulbine abyssinica* + *Withania somnifera*	Roots	Infusion, oral	Internal sores	(76)
3	*Withania somnifera* + *Helichrysum caespititium* + *Solanum campylacanthum*	Tuber + roots	Decoction, oral	Pain	(76)
3	*Withania somnifera* + *Solanum lichtensteinii* + *Bulbine abyssinica*	Tuber + roots	Infusion, oral	Internal sores	(76)
4	*Dicoma galpinii* + *Ziziphus zeyheriana* + *Senna italica* + *Cadaba aphylla*	Roots	Decoction. Oral	Pains	(76)
4	*Grewia occidentalis* + *Olea europaea* + *Zanthoxylum capense* + *Aloe ferox*	Leaves + sap	Infusion	Gallsickness	(59)
5	*Drimia sanguinea* + *Terminalia sericea* + *Senna italica* + *Elephantorrhiza elephantina* + *Jatropha zeyheri*	Bulb + roots + roots+ bulb + bulb	Poultice, topical	Anemia	(12)
6	*Dicerocaryum senecioides* + *Drimia sanguinea* + *Pouzolzia mixta* + *Peltophorum africanum* + *Senna italica* + *Hypoxis hemerocallidea*	Whole plant + bulb + roots + leaves + bulb + bulb	Poultice, topical	Flea eradication	(12)

### Method of Preparing Medicinal Plants for the Treatment of Cattle Diseases

**Figure 4:**
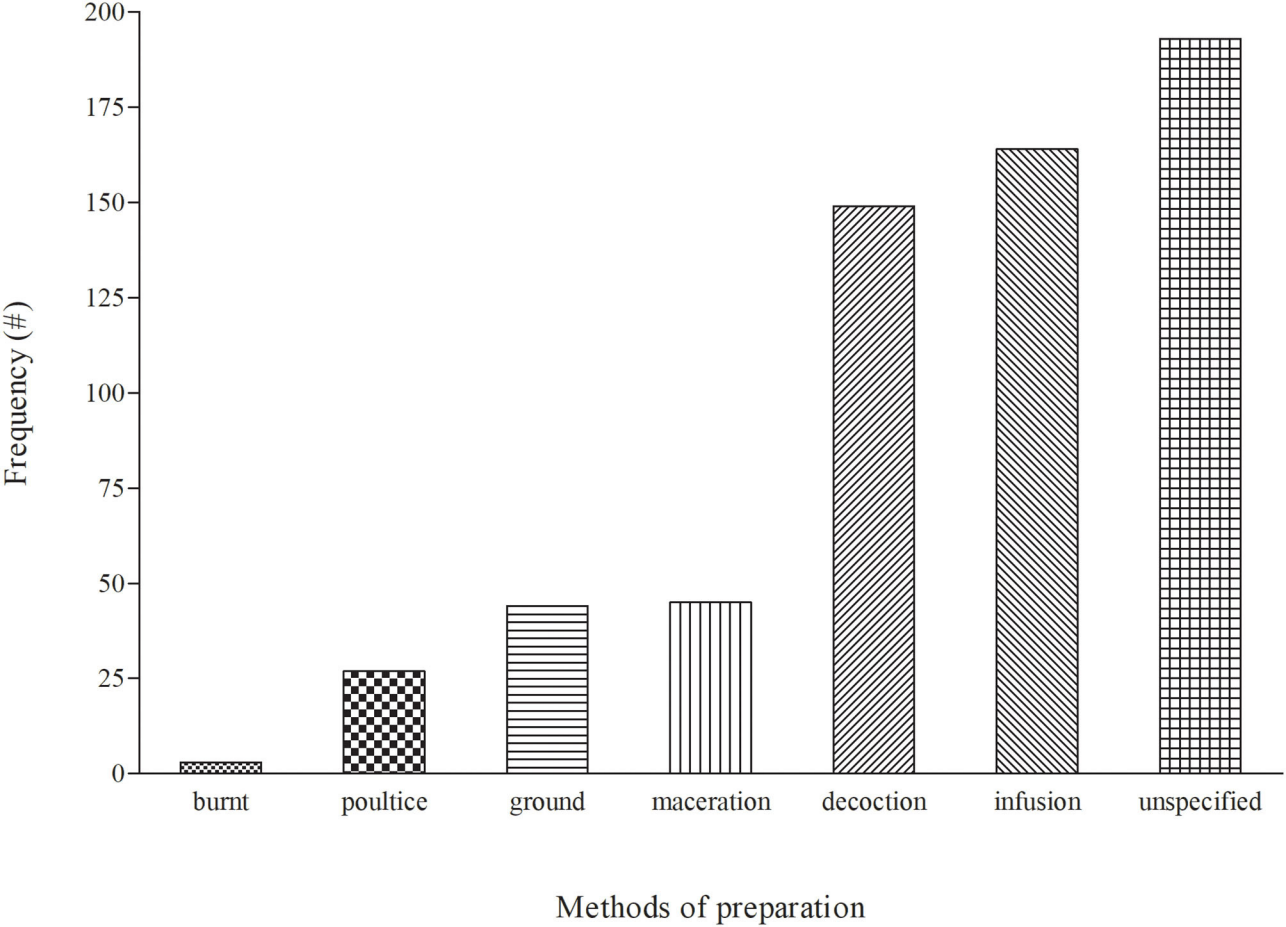
Distribution of the modes of preparation for medicinal plants used to treat cattle diseases in South Africa.

Before administration of medicinal plants to treat cattle diseases, diverse methods of preparation are utilized, which may differ depending on the location and culture. Six (6) preparation methods (burnt, decoction, ground, infusion, maceration, and poultice) were used for treating diseases in cattle (Figure 4). Infusion (166 = 27.25%) was a popular method and it involves pouring cold/hot/warm water onto the plant material and allowing the mixture to cool. This was followed by decoction (149 = 24.46%), which involved boiling plant materials in a specific amount of water and allowing the mixture to cool before administration. However, the current observation differs from other countries whereby crushing and pounding were the most common used preparation methods for livestock diseases (101–103). Other methods of preparation such as maceration, grinding and poultice had low frequencies in the range of 4–7%. The methods of preparation differ depending on the type of disease being treated and the site of the ailment. The majority of the preparations were made using water.

### Mode of Administration/Application of Medicinal Plants for the Treatment of Cattle Diseases

**Figure 5:**
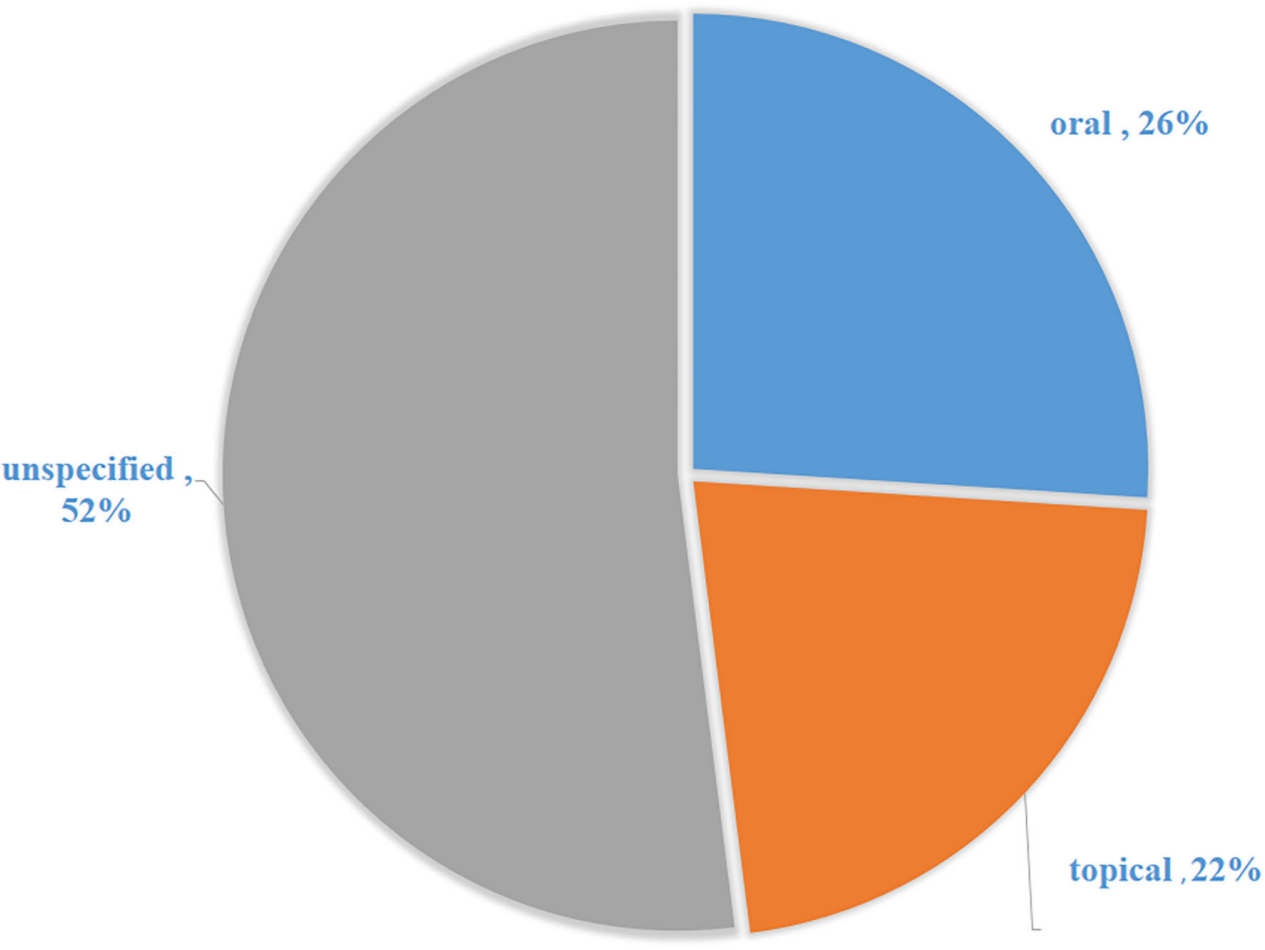
Distribution of the mode of administration for medicinal plants used to treat cattle diseases in South Africa.

The local communities use a variety of methods to administer EVM plants when treating diseases in cattle (Figure 5). The major route of administration for EVM plants was oral-based (157 = 26.5%). Oral administration is a simple and non-invasive form of systemic treatment. The route allows for the rapid absorption and distribution of the prepared medicines and allowing for sufficient curative power to be delivered (104). Topical which contributed 21.8% (105) was the second widely mode of application while 51.9% (308) of cases did not specify how herbal remedies should be administered. Across many African cultures, oral administration of medicinal plants is the most common route used to treat disease in cattle, as this ensures fast and direct interaction with different plant compounds at the site of action (101, 106, 107). The majority of the research documented in the current review omitted the dosage and vehicle usage. The dosage is important because it indicates how much should be used to treat the cattle and the units of measurement. However, EVM are generally known to have a significant flaw in terms of accuracy and standardization (102, 108).

### Common Diseases in Cattle Treated With Plants and Associated Indigenous Knowledge

**Figure 6:**
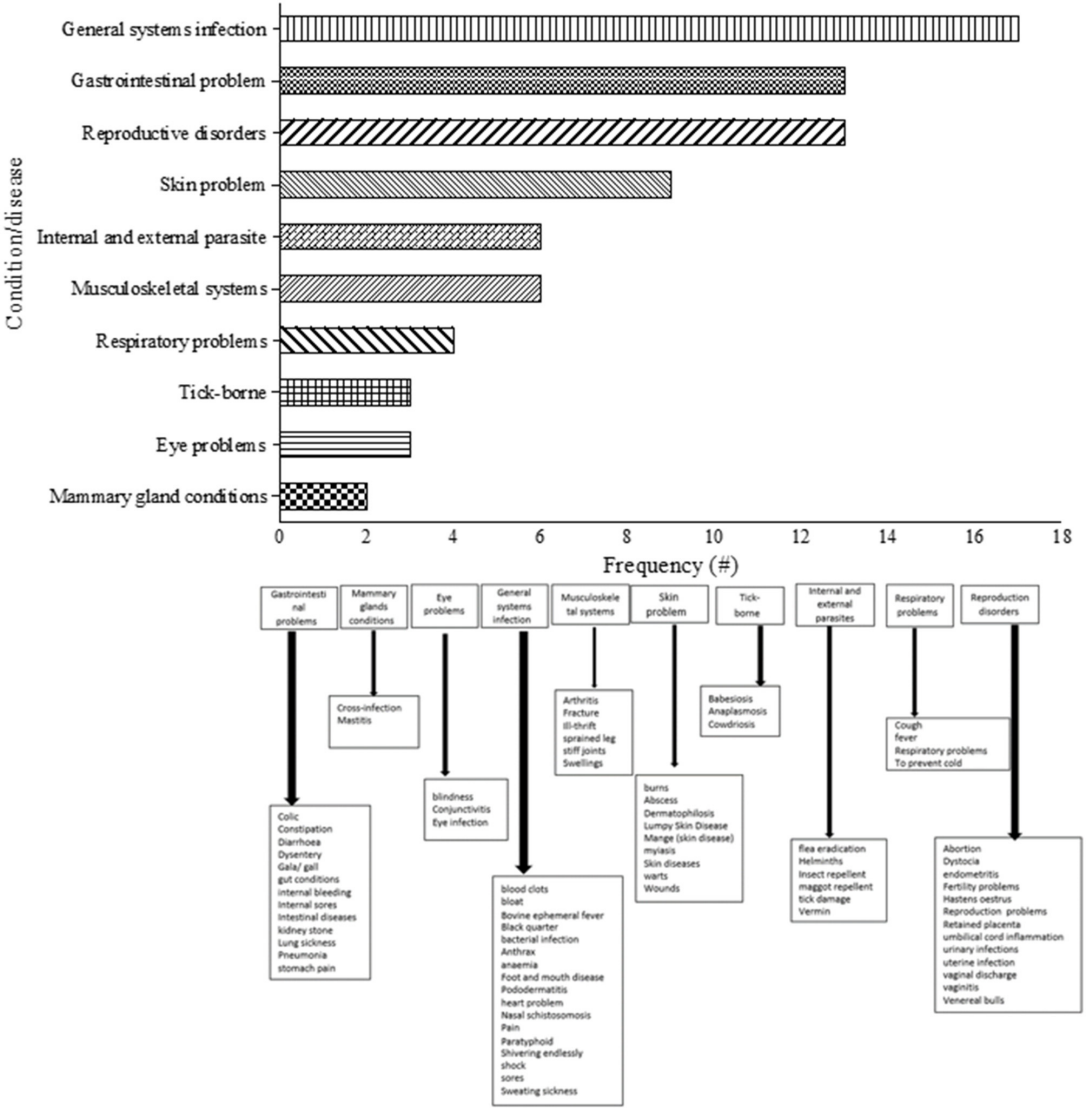
An overview of common cattle ailments treated with medicinal plants in South Africa.

A total of 310 medicinal plants were used to treat several diseases in cattle which were categorized into 10 major groups (Figure 6). The classification of the different diseases was based on the study by Ndou (76), with slight modification. Some of the dominant categories included general systems infection, reproduction disorders, gastrointestinal problems, skin problem, internal/external parasites, musculoskeletal systems, and respiratory problems. On the other hand, treatment of conditions such as eye problems, tick-borne and mammary glands problem were relatively lower in terms of mentions in the reviewed literature. General systems infection was regarded as the most common disease category in cattle (Supplementary Table 3). The majority of these health challenges including digestive problems were easily diagnosed by participants through observation which may explain their high degree of mentions (30, 33–37). The current review identified that the 9 common conditions were anaplasmosis (treated with 69 plants), retained placenta and wounds (treated with 59 plants), diarrhea (treated with 50 plants), babesiosis (treated with 47 plants), helminths (treated with 46 plant) and constipation (treated with 25 plants). Plants such as *Drimia sanguinea* (Schinz) Jessop, *Elephantorrhiza elephantina* (Burch.) Skeels, *Senna italica* Mill., *Boophone disticha* (L.f.) Herb., *Dicerocaryum eriocarpum* (Decne.) Abels, *Aloe ferox* Mill., *Cassia abbreviata* Oliv., *Cussonia spicata* Thunb., and *Cissus quadrangularis* L. were recorded as the most frequently mentioned ones for treating cattle diseases (Supplementary Table 3).

Given that the incidence and severity of various cattle diseases are widespread in rural areas (109–112), the detrimental effect on meat and milk production are often enormous on small-holder livestock farmers (11, 30, 42, 46). As a result, indigenous communities extensively depend on the use of EVM and associated indigenous knowledge to understand the cause, clinical signs and transmission mode of disease occurrence (39, 113). The ability of the community members to understand the diseases is achieved through experiences. They use techniques such as observing the breathing and vocalization, urine and dung, tasting milk, behavioral change, knowledge of vectors and social interaction (76, 114).

### Overview of Biological Evaluation of Plants Used to Manage Cattle Diseases

Out of the 310 plants, ~21% (66 plants) have been screened for biological activity in targeted assays relating to EVM used against cattle diseases (Table 3). Plants were tested for biological activities including antibacterial, antifungal, anti-ticks, antioxidant, antimycobacterial, anti-inflammatory and cytotoxicity. An estimated 70% of the plants (46 of the 66) were screened for antibacterial activity which make it the most studied biological activity. In addition, 51% of the plants (34 of the 66) were evaluated for anthelmintic property while 38% (25 of the 66 plants) have been tested for safety based on cytotoxicity effect. The most frequently screened plant was *Aloe marlothii* that have been screened in 11 bioassays. Other plants that have been subjected to multiple bioassays were *Cissus quadrangularis, Dicerocaryum eriocarpum, Schkuhria pinnata*, and *Volkameria glabra* (7 bioassays), *Ricinus communis* and *Schotia brachypetala* (6 bioassays), *Aloe ferox, Apodytes dimidiata, Clausena anisata, Cussonia spicata, Elephantorrhiza elephantina, Pterocarpus angolensis, Sclerocarya birrea, Zanthoxylum capense*, and *Ziziphus mucronata* (5 bioassays). Plants used for therapeutic purposes are normally assumed to be safe. This is mainly due to the long-term use of medicinal plants for the treatment of diseases based on basic knowledge accumulated and shared from generation to generation over many centuries (136).

**Table 3:** Overview of the biological evaluation of plants used to manage cattle diseases in South Africa.

**Scientific name**	**Screened activity (Reference)**	**Number of Assays conducted**
*Aloe marlothii* A.Berger	Antibacterial, Antifungal, Antimycobacterial, and Cytotoxicity (78) Antibacterial, Anti-inflammatory, and Mutagenicity (115) Antibacterial, Anti-rickettsial, Anti-babesial, and Antioxidant (116) Anti-ticks and Toxicity (117)	11
*Aloe arborescens* Mill.	Antibacterial and cytotoxicity (118)	2
*Aloe ferox* Mill.	Anti-parasitic (73) Anti-ticks and Toxicity (117) *In vitro* and *in vivo* acaricidal (65) Anthelminthic (119) Anthelminthic (120) Anti-ticks (121)	5
*Apodytes dimidiata* E.Mey. ex Arn.	Antiparasitic, Antibacterial, Antioxidant, Cytotoxicity, and Antifungal (122)	5
*Balanites maughamii* Sprague	Antibacterial (64)	1
*Bauhinia thonningii* Schum. (Sny: *Piliostigma thonningii* (Schum.) Milne-Redh.)	Antibacterial (64)	1
*Bolusanthus speciosus* (Bolus) Harms	Antibacterial and Cytotoxicity (123)	2
*Breonadia salicina* (Vahl) Hepper & J.R.I.Wood	Antibacterial (64)	1
*Calpurnia aurea* (Aiton) Benth.	Anthelmintic and cytotoxicity (124) Acaricidal and cytotoxicity (105, 125, 126) Antibacterial and Cytotoxicity (123) Acaricidal (127)	3
*Cassia abbreviata* Oliv.	Anthelmintic (69)	1
*Cissus quadrangularis* L.	Anthelmintic and cytotoxicity (124) Acaricidal and cytotoxicity (105, 125, 126) Antibacterial, Anti-inflammatory, and Mutagenicity (115) Antibacterial, Anthelmintic and toxicity (128) Acaricidal (127)	7
*Clausena anisata* (Willd.) Hook.f. ex Benth.	Antiparasitic, antibacterial, antioxidant, cytotoxicity, and antifungal (122)	5
*Coddia rudis* (E.Mey. ex Harv.) Verdc.	Antibacterial (68)	1
*Combretum caffrum* (Eckl. & Zeyh.) Kuntze	Antibacterial and Antifungal (129)	2
*Curtisia dentata* (Burm.f.) C.A.Sm.	Anthelmintic (130)	1
*Cussonia spicata* Thunb.	Antibacterial, Anti-inflammatory, and Mutagenicity (115) Antibacterial, Anthelmintic and toxicity (128)	5
*Cynanchum viminale* (L.) L. (Syn: *SarcoStemma viminale* (L.) R.Br.)	Antibacterial, Anti-inflammatory, and Mutagenicity (115)	3
*Dicerocaryum eriocarpum* (Decne.) Abels	Anti-parasitic (73) Anthelmintic (120) Antibacterial, Anti-inflammatory, and Mutagenicity (115) Antibacterial, Anthelmintic and toxicity (128)	7
*Dombeya rotundifolia* (Hochst.) Planch.	Antibacterial (64) Antibacterial, Anthelmintic and toxicity (128)	3
*Drimia sanguinea* (Schinz) Jessop Syn: *Urginea sanguinea* Schinz	Antibacterial, Anti-rickettsial, Anti-babesial, and Antioxidant (116)	4
*Elephantorrhiza elephantina* (Burch.) Skeels(Syn: *Acacia Elephantorrhiza*)	Antibacterial, Anti-rickettsial, Anti-babesial, and Antioxidant (116) Anthelminthic (119)	5
*Elephantorrhiza obliqua* Burtt Davy	Antibacterial, Antifungal, Antimycobacterial, and Cytotoxicity (78)	4
*Gardenia volkensii* K.Schum.	Anti-parasitic (73) Anthelmintic (120)	2
*Gnidia capitata* L.f.	Antibacterial, Anthelmintic and toxicity (128)	3
*Harpephyllum caffrum* Bernh.	Antibacterial (68)	1
*Helichrysum caespititium* (DC.) Sond. ex Harv.	Anthelmintic (120)	1
*Helichrysum kraussii* Sch.Bip.	Anti-parasitic (73)	1
*Heteromorpha arborescens* (Spreng.) Cham. & Schltdl.	Antibacterial and cytotoxicity (123)	2
*Hippobromus pauciflorus* Radlk.	Antibacterial, Anthelmintic and toxicity (128)	3
*Hyperacanthus amoenus* (Sims) Bridson	Antibacterial (64)	1
*Hypoxis rigidula* Baker	Anthelmintic and cytotoxicity (124) Acaricidal and cytotoxicity (105, 126)	3
*Jatropha curcas* L.	Anti-ticks and toxicity (117)	2
*Jatropha zeyheri* Sond.	Antibacterial, Anti-inflammatory, and Mutagenicity (115)	3
*Lantana camara* L.	*In vitro* and *in vivo* acaricidal (65)	1
*Leonotis leonurus* (L.) R.Br.	Anthelminthic (119) Anti-ticks (121)	2
*Maerua angolensis* DC.	Anthelmintic and cytotoxicity (124) Acaricidal and cytotoxicity (105, 126)	3
*Melia azedarach* L.	Antiparasitic, antibacterial, antioxidant, cytotoxicity, and antifungal (122)	4
*Pappea capensis* Eckl. & Zeyh.	Anthelmintic (120, 131)	1
*Pelargonium luridum* (Andrews) Sweet	Anthelmintic and cytotoxicity (124) Acaricidal and cytotoxicity (105, 126) Acaricidal (127)	3
*Peltophorum africanum* Sond.	Antioxidant, antibacterial, anthelmintic and toxicity (132) Anthelmintic (69)	4
*Pittosporum viridiflorum* Sims	Antibacterial and cytotoxicity (123)	2
*Plumbago zeylanica* L.	Antiviral and cytotoxicity (133)	2
*Pouzolzia mixta* Solms	Antibacterial, Anthelmintic and toxicity (128)	3
*Ptaeroxylon obliquum* (Thunb.) Radlk.	*In vitro* and *in vivo* acaricidal (65)	1
*Pterocarpus angolensis* DC.	Antibacterial, Anti-inflammatory, and Mutagenicity (115) Antibacterial, Anthelmintic, and toxicity (128)	5
*Rhoicissus tridentata* (L.f.) Wild & R.B.Drumm.	Antibacterial, Anti-rickettsial, Anti-babesial, and Antioxidant (116)	4
*Ricinus communis* L.	Anti-ticks and toxicity (117) Antibacterial, Anti-inflammatory, and Mutagenicity (115) Antibacterial, Anthelmintic and toxicity (128)	6
*Salix capensis* Thunb.2	Antibacterial and Antifungal (129)	
*Schkuhria pinnata* (Lam.) Kuntze ex Thell.	Anthelmintic and cytotoxicity (124) Acaricidal and cytotoxicity (105, 125, 126) Antibacterial, Anti-inflammatory, and Mutagenicity (115) Acaricidal (127)	7
*Schotia brachypetala* Sond.	Antibacterial, antifungal, Antimycobacterial, Cytotoxicity (78) Antibacterial, Anthelmintic and toxicity (128) Anthelmintic (69)	6
*Schotia latifolia* Jacq.	Antibacterial and Antifungal (129)	2
*Sclerocarya birrea* (A.Rich.) Hochst.	Anthelmintic and cytotoxicity (124) Acaricidal and cytotoxicity (105, 125, 126) Antibacterial, Anthelmintic and toxicity (128) Acaricidal (127)	5
*Searsia lancea* (L.f.) F.A. Barkley (Syn: *Rhus lancea* L.f.)	Antibacterial, Anthelmintic and toxicity (128)	3
*Secamone filiformis* J.H. Ross	Antibacterial, Anthelmintic and toxicity (128)	3
*Senecio barbertonicus* Klatt	Anthelmintic (120)	1
*Senna italica* Mill.	Anthelmintic and cytotoxicity (124) Acaricidal and cytotoxicity (105, 125, 126) Anti-tick (134) Acaricidal (127) Anthelmintic (69)	4
*Synadenium cupulare* L.C. Wheeler	Antibacterial, Anthelmintic and toxicity (128)	3
*Tabernaemontana elegans* Stapf	Anthelmintic and cytotoxicity (124) Acaricidal and cytotoxicity (105, 125, 126) Acaricidal (127)	3
*Tagetes minuta* L.	*In vitro* and *in vivo* acaricidal (65) Anti-ticks (135)	2
*Tephroseris palustris* (L.) Rchb. (Syn: *Senecio congestus* (R.Br.) DC.)	Anthelmintic (120)	1
*Tetradenia riparia* (Hochst.) Codd	Antibacterial and cytotoxicity (118)	2
*Trema orientalis* (L.) Blume	Antibacterial and cytotoxicity (118)	2
*Vachellia nilotica* (L.) P.J.H. Hurter & Mabb. (Syn: *Acacia nilotica* (L.) Delile)	Antibacterial and cytotoxicity (118)	2
*Volkameria glabra* (E.Mey.) Mabb. & Y.W. Yuan (Syn: *Clerodendrum glabrum* E.Mey.)	Anti-ticks and toxicity (117) Antiparasitic, antibacterial, antioxidant, cytotoxicity, and antifungal (122)	7
*Zanthoxylum capense* (Thunb.) Harv.	Antiparasitic, antibacterial, antioxidant, cytotoxicity, and antifungal (122)	5
*Ziziphus mucronata* Wild.	Antibacterial, anti-inflammatory and Mutagenicity (122) Antibacterial, anthelmintic and toxicity (128)	5

## Concluding Remarks and Future Perspective

Based on this extensive review, South Africa has a diverse range of plants used for mitigating diseases affecting cattle. The distribution and utilization pattern of EVM reveals a significant variation across a range of geographical settings for 7 out of the 9 provinces in South Africa. Despite the gradual socio-cultural transformation over the years, the inhabitants have retained remarkable knowledge of the plants and their uses up to present days. This suggests that the use of plants for the management of cattle diseases remain culturally rooted among South Africans. The leaves were the most commonly used plant part while the most common methods of preparation were infusions and decoctions. Even though we successfully generated an inventory of 310 medicinal plants used to treat cattle diseases, significant knowledge gaps such as the absence of diagnostic methods for the diseases, preparation methods, administration route and plant parts existed for a number of the plants. This fragmented information emphasizes the need for a well-planned and holistic approach when conducting EVM surveys. The need to adhere to good practices and guidelines particularly “The recommended standards for conducting and reporting ethnopharmacological field studies” (137) and “The need for accurate scientific nomenclature for plants” cannot be overemphasized (138). Furthermore, documenting the use of plants in EVM among South African ethnic groups should embrace indigenous research methodologies in order to gain more cultural insight from the participants. South Africa's unique heritage, both in terms of its rich plant diversity and its cultural traditions, need to be studied, and developed for the benefit of all its people and animals. Furthermore, pharmacological properties studies of EVM plants are a worthwhile endeavor that can contribute to the discovery of new entity to existing drug pools. Establishment of the mechanisms of action remain pertinent to mitigate the drug resistance issues that is increasingly encountered among disease-causing organisms. Toxicology studies must also be strongly incorporated so that potential toxic effects of plants can be identified at early stage of bio-prospecting. In addition, the study of the synergistic effects of plants used in combination would also be beneficial in the development of potent extracts or herbal mixture for resource-poor livestock farmers.

## Author Contributions

The project was conceptualized by MVC with guidance from AOA and MM. MVC prepared the draft manuscript under the supervision of AOA and MM. All authors contributed to the article and approved the submitted version.

## Author Disclaimer

Any opinion, finding, conclusion or recommendation expressed in this material is that of the authors and the NRF (Funder) does not accept any liability in this regard.

## Conflict of Interest

The authors declare that the research was conducted in the absence of any commercial or financial relationships that could be construed as a potential conflict of interest.

## Publisher's Note

All claims expressed in this article are solely those of the authors and do not necessarily represent those of their affiliated organizations, or those of the publisher, the editors and the reviewers. Any product that may be evaluated in this article, or claim that may be made by its manufacturer, is not guaranteed or endorsed by the publisher.
