# Supplementary material for: Ethnoveterinary Knowledge and Biological Evaluation of Plants Used for Mitigating Cattle Diseases: A Critical Insight Into the Trends and Patterns in South Africa
Source: Front Vet Sci. 2021 Aug 19;8:710884. doi: 10.3389/fvets.2021.710884 (PMC8417044; doi:10.3389/fvets.2021.710884)
Supplement: Supplementary file 1 [file Table_1.pdf]

Supplementary Table S1: An inventory of plants used against cattle diseases across different provinces of South Africa. The botanical name and families were verified using the Plant list (http://www.theplantlist.org) and plantZAfrica (http://pza.sanbi.org/); #Local name, S = Sesotho, B = Setswana, P = Sepedi, V = Venda, X = Xhosa, Z = Zulu, T = Tsonga, SS = Swati, Eng.= English, AF = Afrikaans

Plants scientific name	Plant family	#Local name	Part used	Preparation method	Administration mode	Diseases	References
Acacia decurrens Willd.	Leguminosae	Indywabasi (X)	Bark	Decoction	Oral	Hastens oestrus	(Masika et al. 2000)
Acacia mearnsii De Wild.	Leguminosae	Indywabasi (X)	Bark	Maceration	Unspecified	Hastens oestrus	(Masika and Afolayan 2003)
Acanthospermum hispidum DC.	Compositae	Muvhavhanyane (V)	Aerial parts	Grinding	Topical	Wounds	(Magwede et al. 2014)
Acokanthera oblongifolia (Hochst.) Benth. & Hook.f. ex B.D.Jacks. (Syn: *Acokanthera oblongifolia* (Hochst.) Codd)	Apocynaceae	Isihlungu (X)	Unspecified	Infusion	Unspecified	Wounds	(Beinart and Brown 2013)
Acokanthera oppositifolia (Lam.) Codd	Apocynaceae	Isihlungusehlathi (X)	Leaves	Infusion	Oral	Paratyphoid	(Mthi et al. 2018)
Acrotome inflata Benth.	Lamiaceae	Mogato (B)	Roots	Decoction	Oral	Wound, Abscess	(Ndou 2018)
Afzelia quanzensis Welw.	Leguminosae	Inkehli (Z)	Unspecified	Unspecified	Unspecified	Unspecified	(Hutchings 1996)
Agapanthus praecox Wild.	Amaryllidaceae	Mavumbula	Roots & Leaves	Infusion	Oral	Black quarter	(Mthi et al., 2018)
Agave americana L.	Agavaceae	Unspecified	Leaves	Maceration	Topical	Wound	(Semenya et al. 2019)
Albizia adianthifolia (Schum.) W.Wight (Albizia sp.)	Leguminosae	Xisitana (T)	Root skin	Infusion	Oral	Swollen stomach	(Khunoana et al. 2019)
Albuca aurea Jacq.	Asparagaceae	Unspecified	Bulb	Unspecified	Unspecified	Helminths	(Kambizi 2014)
		Unspecified	Unspecified	Unspecified	Unspecified	Helminths	(Gerstner 1938)
Alepidea amatymbica Eckl. & Zeyh.	Apiaceae	Unspecified	Bulb	Unspecified	Unspecified	Constipation, helminths	(Kambizi, 2014)
Allium cepa L.	Amaryllidaceae	Unspecified	Bulb	Maceration	Oral	Skin problems, cowdriosis	(Semenya et al., 2019)
Aloe marlothii A.Berger	Xanthorrhoeaceae	Seema ka maoto (P)	Leaves	Unspecified	Unspecified	Constipation	(Mongalo and Makhafola 2018)
		Mahgana(T)	Leaves	Infusion	Oral	Unspecified	(Khunoana et al., 2019)
		Mokgopa (B)	Leaves	Unspecified	Unspecified	Gall, diarrhoea	(Van der Merwe et al. 2001)

Plants scientific name	Plant family	#Local name	Part used	Preparation method	Administration mode	Diseases	References
Aloe arborescens Mill.	Xanthorrhoeaceae	Unspecified	Leaves	Decoction	Unspecified	Anaplasmosis, parasites, diarrhoea, constipation, Retained placenta, dystocia, maggots	(Hutchings et al. 1996)
Aloe ferox Mill.	Xanthorrhoeaceae	Ikahala (X)	Unspecified	Infusion	Unspecified	Unspecified	(Beinart & Brown, 2013)
		Lekhala (S)	Leaves	Decoction	Unspecified	Anaplasmosis	(Beinart & Brown, 2013)
		Ikhala (X)	Leaves	Decoction	Oral	Anaplasmosis, constipation	(Mthi et al. 2020)
		IKhala (X)	Leaves	Infusion	Topical	Babesiosis	(Moyo,2008)
		Ikhala (X)	Leaves	Infusion & Grinding	Topical	Ticks	(Soyelu& Masika, 2009)
		IKhala (X)	Leaves	Infusion	Unspecified	Wounds	(Masika & Afolayan, 2003)
		iKhala (X)	Leaves	Decoction	Oral	Anaplasmosis, babesiosis, helminths	(Dold & Cocks, 2001)
		Unspecified	Leaves	Maceration	Topical	Babesiosis	(Semenya et al., 2019)
		Inhlaba (SS)	Leaves	Infusion	Oral	Ticks	(Shiba 2018)
Aloe greatheadii Schönland	Xanthorrhoeaceae	Kgopane e nyane (B)	Leaves	Decoction	Oral	Helminths	(Moichwanetse et al, 2020)
		Kgophane (B)	Whole plant	Unspecified	Unspecified	Retained placenta, diarrhoea	(Van der Merwe et al., 2001)
Aloe hahnii Gideon F.Sm. & Klopper	Xanthorrhoeaceae	Tshikhophatshiṱuku (V)	Roots/ Leaves	Decoction /Infusion	Unspecified	Burns, helminths, conjunctivitis	(Ramovha & van Wyk, 2016)
Aloe maculata All.	Xanthorrhoeaceae	Unomaweni (X)	Leaves	Decoction	Oral	Babesiosis	(Mthi et al. 2020)

Plants scientific name	Plant family	#Local name	Part used	Preparation method	Administration mode	Diseases	References
		Unspecified	Leaves	Infusion	Unspecified	Cowdriosis	(Hutchings et al. 1996)
Aloe spicata L.f.	Xanthorrhoeaceae	Kgophane/Mokgopa (B)	Leaves	Infusion	Oral	Diarrhoea (Blood scours)	(Beinart & Brown, 2013)
Aloe tenuior Haw.	Xanthorrhoeaceae	uMjingqa (X)	Leaves	Decoction	Oral	Anaplasmosis, constipation, helminths, fertility problem, anthrax, sores	(Dold & Cocks, 2001)
Aloe vera (L.) Burm.f.	Xanthorrhoeaceae	Unspecified	Leaves	Maceration	Topical	Retained placenta	(Semenya et al., 2019)
		Sekgopha (P)	Leaves	Unspecified	Unspecified	Wounds	(Matlebyane et al., 2010).
		Kgopane ya thaba (B)	Leaves	Decoction	Oral	Hastens oestrus	(Moichwanetse et al, 2020)
Aloe zebrina Baker	Xanthorrhoeaceae	kgopsana (P)	Unspecified	Unspecified	Unspecified	Retained placenta, diarrhoea, gala	(Matlebyane et al., 2010).
		Kgophane (B)	Whole plant	Unspecified	Unspecified	Wounds	(Van der Merwe et al., 2001)
		Kgophane (B)	Roots/ Leaves	Decoction /Burn	Oral	Burns, Helminths, conjunctivitis	(Ndou, 2018)
		Chovoloti (T)	Leaves	Grinding	Topical	Retained placenta, abscess	(Luseba & Van der Merwe, 2006).
		Kgopha (P)	Leaves	Grinding	Topical	Wounds	(Mogale, 2017).
Amaranthus blitum L. (Syn: *Amaranthus lividus* L)	Amaranthaceae	Modinakana (B)	Leaves	Infusion	Oral	Wounds	(Ndou, 2018)
Ammocharis coranica (Ker Gawl.) Herb.	Amaryllidaceae	icukudo, incotho (Z)	Unspecified	Unspecified	Unspecified	Wounds	(Hutchings, 1996)
Apodytes dimidiata E.Mey. ex Arn.	Icacinaceae	Umdakana (Z)	Bark & Leaves	Unspecified	Unspecified	Unspecified	(Hutchings, 1996)
		Unspecified	Bark	Unspecified	Unspecified	Helminths	(Gerstner, 1938)

Plants scientific name	Plant family	#Local name	Part used	Preparation method	Administration mode	Diseases	References
Artemisia afra Jacq. ex Willd.	Compositae	Lengana (B)	Leaves	Infusion	Oral	Constipation	(Ndou, 2018)
Asparagus virgatus Baker (Syn: *Protasparagus virgatus* (Baker) Oberm.)	Asparagaceae	Unspecified	Roots	Decoction / Infusion	Unspecified	Cough	(Watt and Breyer-Brandwijk 1962)
Asparagus africanus Lam.	Asparagaceae	Unspecified	Bulb	Unspecified	Unspecified	Helminths	(Kambizi, 2014)
		Lufhaladzama kole (V)	Whole plants	Infusion	Oral	Cowdriosis	(Luseba & Tshisikhawe, 2013).
Asparagus falcatus L.	Asparagaceae	Unspecified	Roots	Decoction	Oral	Constipation	(Semenya et al., 2019)
Asparagus laricinus Burch.	Asparagaceae	lesitwane (B)	Tubers	Unspecified	Unspecified	Constipation	(Van der Merwe et al., 2001)
Asparagus nodulosus (Oberm.) J.-P.Lebrun & Stork	Asparagaceae	Radopolwane/ polopolwane (B)	Roots	Maceration	Topical	Babesiosis, uterine infection, umbilical cord inflammation	(Ndou, 2018)
Asparagus setaceus (Kunth) Jessop	Asparagaceae	iMvane (X)	Roots	Infusion/ Decoction	Unspecified	Eye infection	(Dold & Cocks, 2001)
Asparagus suaveolens Burch.	Asparagaceae	lesitwane (B)	Tubers	Unspecified	Unspecified	Retained placenta	(Van der Merwe et al., 2001)
Azima tetracantha Lam.	Salvadoraceae	iGceleya (X)	Roots	Infusion	Unspecified	Babesiosis, uterine infection, umbilical cord inflammation	(Dold & Cocks, 2001)
Balanites maughamii Sprague	Zygophyllaceae	Unspecified	Leaves	Decoction	Unspecified	Dystocia	(Mahlo, 2006)
		Nulu (T)	Leaves	Infusion	Oral	Diarrhoea	(Luseba & Van der Merwe, 2006).
Baphia racemosa (Hochst.) Baker	Leguminosae	Isifithi (X)	Unspecified	Infusion	Unspecified	Diarrhoea	(Beinart & Brown, 2013)
Bauhinia thonningii Schum. (Sny: *Piliostigma thonningii* (Schum.) Milne-Redh.)	Leguminosae	Mutulume (V)	Bark	Maceration	Unspecified	Anaplasmosis	(Mahlo, 2006)
Bersama tysoniana Oliv.	Melianthaceae	Isindiyandiya (X)	Bark	Decoction	Unspecified	Diarrhoea	(Masika & Afolayan, 2003)
		Unspecified	Bark	Decoction	Unspecified	Anaplasmosis	(Watt and Breyer-Brandwijk 1962)

Plants scientific name	Plant family	#Local name	Part used	Preparation method	Administration mode	Diseases	References
Bolusanthus speciosus (Bolus) Harms	Leguminosae	Nkohlwane (V)	Roots	Grounding	Oral	Anaplasmosis	(Luseba & Tshisikhawe, 2013).
Boophone disticha (L.f.) Herb.	Amaryllidaceae	iShwadi (X)	Bulb	Decoction	Unspecified	Retained placenta	(Dold & Cocks, 2001)
		InCwadi (X)	Bulb	Decoction	Unspecified	Babesiosis	(Masika & Afolayan, 2003)
		Leshoma/Leswama (B)	Bulb	Decoction	Oral/ Topical	Babesiosis	(Ndou, 2018)
		Mathubadifala (B)	Unspecified	Unspecified	Unspecified	Retained placenta, abortion, fracture	(Getchell et al 2001)
		Lesoma/Legwama (B)	Leaves & Roots	Maceration	Oral	Anaplasmosis	(Moichwanetse et al, 2020)
		Matubadifala (B)	Bulb scales	Unspecified	Unspecified	Retained placenta, wounds	(Van der Merwe et al., 2001)
		Mathubadifala (B)	Leaves	Grinding	Unspecified	Abortion	(Beinart & Brown, 2013)
Brachylaena elliptica (Thunb.) Less.<br>(Sny: *Brachylaena elliptica* (Thunb.) DC.)	Compositae	Unspecified	Unspecified	Unspecified	Unspecified	Fertility problems, venereal bulls	((Gerstner, 1939))
Brachylaena discolor DC.	Compositae	Unspecified	Leaves	Infusion	Unspecified	Unspecified	(Hutchings et al. 1996)
Breonadia salicina (Vahl) Hepper & J.R.I.Wood	Rubiaceae	Mutulume (V)	Bark	Maceration	Unspecified	Helminths	(Mahlo, 2006)
Bridelia micrantha (Hochst.) Baill.	Phyllanthaceae	Incinci (Z)	Bark	Infusion	Unspecified	Intestinal diseases, retained placenta	(Hutchings, 1996)
Bulbine latifolia (L.f.) Spreng.<br>(Sny: *Bulbine latifolia* (L.f.) Roem. Et Schult.)	Xanthorrhoeaceae	Unspecified	Roots	Unspecified	Unspecified	Nasal schistosomosis	(Kambizi, 2014)
		Unspecified	Leaves	Infusion	Topical & Oral	Skin diseases	(Semenya et al., 2019)
Bulbine abyssinica A.Rich.	Xanthorrhoeaceae	Makgabenyana (B)	Unspecified	Unspecified	Unspecified	Ticks, helminths	(Getchell et al 2001)

Plants scientific name	Plant family	#Local name	Part used	Preparation method	Administration mode	Diseases	References
		Makgabenyana (B)	Roots	Infusion	Oral	Anaplasmosis	(Ndou, 2018)
Bulbine alooides (L.) Willd.	Xanthorrhoeaceae	IRooiwater (AF)	Roots	Decoction	Unspecified	Internal sores	(Dold & Cocks, 2001)
Bulbine asphodeloides (L.) Spreng.	Xanthorrhoeaceae	Unspecified	Unspecified	Unspecified	Unspecified	Babesiosis	(Hutchings et al. 1996)
Bulbine frutescens (L.) Willd.	Xanthorrhoeaceae	Balsemkopiva (X)	Unspecified	Infusion	Unspecified	Unspecified	(Beinart & Brown, 2013)
Burchellia bubalina (L.f.) Sims	Rubiaceae	Unspecified	Leaves	Unspecified	Unspecified	Anaplasmosis	(Kambizi, 2014)
Cadaba aphylla (Thunb.) Wild	Capparaceae	Munnamutsu (S)	Roots	Decoction	Oral	Cowdriosis	(Ndou, 2018)
Callilepis laureola DC.	Compositae	ihlamvu, impila (Z)	Roots	Poultice	Unspecified	Pains (from sores and fractures)	(Hutchings, 1996)
		Unspecified	Roots	Poultice	Unspecified	Maggots	(Watt and Breyer-Brandwijk 1962)
Calpurnia aurea (Aiton) Benth.	Leguminosae	Insiphane (Z)	Unspecified	Unspecified	Unspecified	Maggots	(Hutchings, 1996)
		Umbethu Umhlahlampet hu (X)	Leaves	Infusion	Topical	Maggots in sores	(Soyelu& Masika, 2009)
		Tlwele (S)	Leaves	Infusion	Unspecified	Maggot-infested wounds	(Beinart & Brown, 2013)
		Unspecified	Unspecified	Unspecified	Unspecified	Diarrhoea	(Bryant 1966)
Capparis sepiaria L.	Capparaceae	Unspecified	Roots	Decoction	Unspecified	Maggots	(Watt and Breyer-Brandwijk 1962)
Capparis tomentosa Lam.	Capparaceae	Unspecified	Roots	Infusion	Unspecified	Anaplasmosis	(Watt and Breyer-Brandwijk 1962)
		Unspecified	Roots	Infusion	Unspecified	Diarrhoea	(Pujol 1990)
		Unspecified	Roots	Decoction	Oral	Diarrhoea	(Semenya et al., 2019)
Carica papaya L.	Caricaceae	Unspecified	Leaves	Decoction	Topical	Diarrhoea	(Semenya et al., 2019)
Carissa spinarum L. (Syn: *Carissa edulis* (Forssk.) Vahl.)	Apocynaceae	Unspecified	Leaves	Maceration	Topical	Ticks	(Semenya et al., 2019)
Carissa bispinosa (L.) Desf. ex Brenan	Apocynaceae	Serokolo (B)	Unspecified	Infusion	Unspecified	Cowdriosis	(Beinart & Brown, 2013)

Plants scientific name	Plant family	#Local name	Part used	Preparation method	Administration mode	Diseases	References
		Tshirungulu (V)	Bulb	Infusion	Oral	Cross-infection	(Luseba & Tshisikhawe, 2013).
Cassia abbreviata Oliv.	Leguminosae	Monepenepe (P)	Bark	Infusion	Unspecified	Dystocia	(Mogale, 2017).
		mulumanamana (V)	Stem/Root Bark	Decoction /Infusion	Unspecified	Helminths	(Ramovha & van Wyk, 2016)
		Unspecified	Bark	Infusion	Topical	Babesiosis	(Semenya et al., 2019)
		Monepenepe (P)	Leaves	Unspecified	Oral	Dermatophilosis	(Mongalo & Makhafola, 2018)
		Muboma (V)	Stem Bark	Infusion	Oral	Unspecified	(Chitura et al. 2018)
		Lumanyama(T)	Bark	Infusion	Oral	Retained placenta	(Luseba & Van der Merwe, 2006).
		Munembenembe (V)	Bark	Grinding	Topical	Helminths	(Magwede et al., 2014)
		Unspecified	Bark	Unspecified	Unspecified	Wounds	(Mphahlele 2016)
Cassine aethiopica Thunb. (Sny: *Mystroxylon aethiopicum* (Thunb.) Loes.)	Celastraceae	uMbomvane (X)	Bark	Decoction	Oral	Helminths	(Dold & Cocks, 2001)
		Unspecified	Bark	Infusion	Unspecified	Cowdriosis	(Watt and Breyer-Brandwijk 1962)
Cassine transvaalensis (Burtt Davy) Codd (Syn: *Elaeodendron transvaalense* (Burtt Davy) R.H.Archer)	Celastraceae	Mulumanama (V)	Fruits	Infusion	Oral	Helminths	(Luseba & Tshisikhawe, 2013).
		Mojelemane (S)	Bark	Unspecified	Unspecified	Helminths	(Van der Merwe et al., 2001)
Cassytha filiformis L.	Lauraceae	Luangalala (V)	Aerial parts	Grounding	Topical	Diarrhoea	(Magwede et al., 2014)
		Luangalala (V)	Stem	Decoction	Oral	Wounds	(Luseba & Tshisikhawe, 2013).
Centella asiatica (L.) Urb.	Apiaceae	Luangalala (V)	Stem	Decoction	Oral	Dystocia	(Moichwanetse et al, 2020)
Cephalanthus natalensis Oliv.	Rubiaceae	Murondo (V)	Leaves	Decoction	Oral	Retained placenta	(Luseba & Tshisikhawe, 2013).
Chlorophytum cremnophilum Van Jaarsv.	Agavaceae	UmKhonzi (X)	Bulb and Flowers	Infusion	Oral	Eye problem	(Mthi & Rust, 2020)
Cissampelos capensis L.f.	Menispermaceae	Unspecified	Roots	Unspecified	Unspecified	Retained placenta	(Kambizi, 2014)

Plants scientific name	Plant family	#Local name	Part used	Preparation method	Administration mode	Diseases	References
Cissus quadrangularis L.	Vitaceae	Mohlabadipoo (P)	Stems	Unspecified	Oral	Skin problems, wounds	(Mongalo & Makhafola, 2018)
		Malongakanya (V)	Branch	Infusion	Oral/ Topical	Unspecified	(Chitura et al., 2018)
		Nyangala(T)	Aerial parts/ Stem	Poultice/ Grinding	Topical/ Oral	Retained placenta, wounds	(Luseba & Van der Merwe, 2006).
		Malongekanye (V)	Aerial parts	Grinding	Topical	Wound, Ticks, lumpy skin disease	(Magwede et al., 2014)
		maswonona; malongekanye; masungunuvhe (V)	Roots/ Leaves	Infusion	Unspecified	Wounds	(Ramovha & van Wyk, 2016)
		Nyangala(T)	Stem	Grinding	Topical	Babesiosis	(Khunoana et al., 2019)
Clausena anisata (Willd.) Hook.f. ex Benth.	Rutaceae	isiFutho umNukambiba (X)	Leaves	Grinding & Decoction	Unspecified	Wounds	(Masika & Afolayan, 2003)
		Umduze (Z)	Unspecified	Unspecified	Unspecified	Foot rot, helminths	(Hutchings, 1996)
Clivia nobilis Lindl. (Clivia sp.)	Amaryllidaceae	Unspecified	Roots	Unspecified	Unspecified	Dysentery	(Kambizi, 2014)
Clutia pulchella L.	Peraceae	uBuhlungu-bedila (X)	Leaves and Stem	Decoction	Unspecified	Stomach problems	(Masika & Afolayan, 2003)
		Umbezo (X)	Unspecified	Unspecified	Unspecified	Anaplasmosis	(Beinart & Brown, 2013)
		Mjamonti (T)	Bark	Decoction	Oral	Paratyphoid	(Khunoana et al., 2019)
		Unspecified	Leaves, Stem, Roots	Infusion	Unspecified	Gall	(Hutchings et al. 1996)
Coddia rudis (E.Mey. ex Harv.) Verdc.	Rubiaceae	Unspecified	Leaves	Unspecified	Unspecified	Pain	(Kambizi, 2014)
Combretum apiculatum Sond. (syn: *Combretum collinum*)	Combretaceae	Muvuvha (V)	Stem Bark	Unspecified	Oral	Ticks	(Chitura et al., 2018)
Combretum caffrum (Eckl. & Zeyh.) Kuntze	Combretaceae	UmDubi (X)	Leaves	Maceration	Topical	Constipation	(Masika et al., 2000)
		UmDubi (X)	Leaves and Bark	Decoction & Grounding	Unspecified	Conjunctivitis	(Masika & Afolayan, 2003)

Plants scientific name	Plant family	#Local name	Part used	Preparation method	Administration mode	Diseases	References
Combretum microphyllum Klotzsch	Combretaceae	Mukopokopo (V)	Roots	Decoction /Infusion	Unspecified	Babesiosis, conjunctivitis	(Ramovha & van Wyk, 2016)
Combretum paniculatum Vent.	Combretaceae	Mpfunta (T)	Root Bark	Decoction	Oral	Babesiosis	(Luseba & Van der Merwe, 2006).
Combretum vendae A.E.van Wyk	Combretaceae	Mugwiti (V)	Leaves	Infusion	Oral	Fertility problems	(Luseba & Tshisikhawe, 2013).
Copaiba mopane (J. Kirk ex Benth.) Kuntze	Leguminosae	Unspecified	Leaves	Decoction	Oral	Gut conditions, diarrhoea, helminths, dystocia	(Semenya et al., 2019)
Crinum stuhlmannii Baker (Syn: *Crinum delagoense* Verdoorn)	Amaryllidaceae	Unspecified	Unspecified	Unspecified	Unspecified	Constipation	(Gerstner, 1939)
		umduze/umduzi (Z)	Unspecified	Unspecified	Unspecified	Unspecified	(Hutchings, 1996)
Crinum moorei Hook.f.	Amaryllidaceae	umduze/ umduzi (Z)	Unspecified	Unspecified	Unspecified	Unspecified	(Hutchings, 1996)
		Unspecified	Unspecified	Unspecified	Unspecified	Unspecified	((Gerstner, 1939))
Croton gratissimus Burch.	Euphorbiaceae	Moologa (B)	Leaves	Grounding	Unspecified	Unspecified	(Ndou, 2018)
		Moologa (B)	Leaves and Roots	Unspecified	Unspecified	Fertility problems	(Van der Merwe et al., 2001)
Cucumis africanus L.f.	Cucurbitaceae	Unspecified	Unspecified	Unspecified	Unspecified	Pneumonia, fertility problem	(Hutchings et al. 1996)
Curtisia dentata (Burm.f.) C.A.Sm.	Curtisiaceae	uMlahleni (X)	Bark	Infusion	Oral	Unspecified	(Dold & Cocks, 2001)
Cussonia spicata Thunb.	Araliaceae	uMsenge (X)	Leaves and bark	Infusion	Oral	Cowdriosis	(Mthi & Rust, 2020)
		uMsenge (X)	Bark and Leaves	Decoction	Unspecified	Retained placenta	(Masika & Afolayan, 2003)
		uMsenge (X)	Bark and Leaves	Infusion	Oral	Babesiosis, anaplasmosis	(Dold & Cocks, 2001)
		uMsenge (X)	Bark	Unspecified	Unspecified	Retained placenta, endometritus, vaginitis	(Kambizi, 2014)

Plants scientific name	Plant family	#Local name	Part used	Preparation method	Administration mode	Diseases	References
		umSenge (X)	Unspecified	Unspecified	Unspecified	Cowdriosis	(Masika et al., 1997)
		UmSenge (X)	Bark	Decoction	Oral	Babesiosis and anaplasmosis	(Masika et al., 2000)
Cyclospermum leptophyllum (Pers.) Sprague	Apiaceae	Lufhelele (V)	Aerial parts	Grinding	Topical	Anaplasmosis	(Magwede et al., 2014)
Cynanchum viminale (L.) L. (Syn: *SarcoStemma viminale* (L.) R.Br.)	Apocynaceae	Neta (T)	Aerial parts	Grinding	Topical	Wounds	(Luseba & Van der Merwe, 2006).
Cyphia stramonium N.E. Br.	Campanulaceae	Unspecified	Leaves	Unspecified	Unspecified	Wound	(Kambizi, 2014)
CyphoStemma natalitium (Szyszyl.) J.J.M.van der Merwe	Vitaceae	Idambiso (X)	Roots	Unspecified	Unspecified	Shivering endlessly	(Hutchings, 1996)
		Unspecified	Unspecified	Unspecified	Unspecified	Colic	((Gerstner, 1939))
CyphoStemma cirrhosum (Thunb.) Desc. ex Wild & R.B.Drumm.	Vitaceae	Unspecified	Stem	Unspecified	Unspecified	Colic	(Kambizi, 2014)
Dalbergia obovata E.Mey.	Leguminosae	Izungu (X)	Leaves	Infusion	Oral	Urinary problems	(Mthi et al., 2018)
Datura stramonium L.	Solanaceae	Unspecified	Leaves	Maceration	Topical	Paratyphoid	(Watt and Breyer-Brandwijk 1962)
Dicerocaryum eriocarpum (Decne.) Abels	Pedaliaceae	Lepate/tshetlho/ Makanangwane (B)	Unspecified	Infusion	Unspecified	Wounds	(Beinart & Brown, 2013)
		Museto (V)	Aerial parts	Infusion	Oral	Retained placenta, anaplasmosis, black quarter, venereal bull	(Luseba & Tshisikhawe, 2013).
		Museto (V)	Leaves and Stem	Infusion	Oral	Dystocia, helminths	(Mabogo 1990)
		Inkundzana (SS)	Leaves	Infusion	Oral	Retained placenta, black quarter	(Shiba, 2018)
		Makanangwane (B)	Roots	Unspecified	Unspecified	Helminths	(Van der Merwe et al., 2001)

Plants scientific name	Plant family	#Local name	Part used	Preparation method	Administration mode	Diseases	References
Dicerocaryum senecioides (Klotzsch) Abels	Pedaliaceae	Tshetlho ya mibitla e mebedi (B)	Whole plant	Poultice	Topical	Retained placenta	(Moichwanetse et al, 2020)
		makangwane (B)	Whole plant	Unspecified	Unspecified	Retained placenta, flea eradication	(Van der Merwe et al., 2001)
		Mompati (P)	Leaves	Infusion	Oral	Dystocia	(Mogale, 2017).
Dichrostachys cinerea (L.) Wight & Arn.	Leguminosae	Moselesele (B)	Roots	Poultice	Topical	Retained placenta	(Moichwanetse et al, 2020)
Dicoma anomala Sond.	Compositae	Hloenya/Loen ya (S)	Whole plant	Infusion	Unspecified	Retained placenta, dystocia, fracture	(Beinart & Brown, 2013)
		Unspecified	Roots	Decoction	Unspecified	Fertility problem, anaplasmosis	(Watt and Breyer-Brandwijk 1962)
Dicoma galpinii F.C.Wilson	Compositae	Tlhlonya (B)	Roots	Infusion / Decoction	Oral	Anaplasmosis	(Ndou, 2018)
Dietes bicolor (Steud.) Sweet ex Klatt	Iridaceae	Mbona kaxam (X)	Roots	Infusion	Oral	Diarrhoea, gala, pains (from sores, fractures), stomach pains	(Mthi et al. 2020)
Dioscorea dregeana (Kunth) T.Durand & Schinz	Dioscoreaceae	Unspecified	Tubers	Unspecified	Unspecified	Anaplasmosis	(Watt and Breyer-Brandwijk 1962)
Dioscorea sylvatica Eckl.	Dioscoreaceae	Unspecified	Tubers	Decoction	Unspecified	Sores and wounds	(Watt and Breyer-Brandwijk 1962)
Diospyros lycioides Desf.	Ebenaceae	Muthala (V)	Leaves	Infusion	Topical	Swollen udder	(Luseba & Tshisikhawe, 2013).
		Muthala (V)	Leaves	Infusion	Topical	Ticks	(Magwede et al., 2014)
Diospyros mespiliformis Hochst. ex A.DC.	Ebenaceae	muţangule (V)	Roots	Infusion	Unspecified	Wounds	(Ramovha & van Wyk, 2016)
Dodonaea viscosa (L.) Jacq.	Sapindaceae	Unspecified	Leaves	Maceration	Topical	Babesiosis	(Semenya et al., 2019)
Dombeya rotundifolia (Hochst.) Planch.	Malvaceae	Unspecified	Leaves	Decoction	Oral	Wounds	(Semenya et al., 2019)

Plants scientific name	Plant family	#Local name	Part used	Preparation method	Administration mode	Diseases	References
		Nsihaphukuma (T)	Leaves	Decoction	Unspecified	Diarrhoea	(Mahlo, 2006)
Drimia altissima (L.f.) Ker Gawl. (Syn: *Urginea altissima* (L.f.) Baker)	Asparagaceae	uZabokwe, iMfilisi (X)	Bulb	Decoction	Oral	Diarrhoea	(Dold & Cocks, 2001)
Drimia elata Jacq. (Syn: *Drimia robusta* Baker)	Asparagaceae	Intongana (X)	Unspecified	Infusion	Unspecified	Intestinal parasites, retained placenta	(Beinart & Brown, 2013)
Drimia sanguinea (Schinz) Jessop (Syn: *Urginea sanguinea* Schinz)	Asparagaceae	Unspecified	Bulb	Maceration	Topical	Babesiosis	(Semenya et al., 2019)
		Sekaname (B)	Bulb	Infusion / Grinding	Oral/ Topical	Wounds	(Beinart & Brown, 2013)
		Sekanama (P)	Bulb	Unspecified	Unspecified	Anthrax, Ticks, sores, black quarter, babesiosis, constipation, helminths, retained placenta	(Mongalo & Makhafola, 2018)
		Sekaname (B)	Unspecified	Unspecified	Unspecified	Unspecified	(Getchell et al 2001)
		Sekaname (B)	Bulb	Poultice	Topical	Anaplasmosis	(Moichwanetse et al, 2020)
		Sekaname (B)	Bulb	Unspecified	Unspecified	Retained placenta, anaemia, helminths	(Van der Merwe et al., 2001)
Dryopteris athamantica (Kunze) Kuntze (Syn: *Nephrodium athamanticum* Hook.)	Dryopteridaceae	Umkhomakhoma (X)	Unspecified	Unspecified	Unspecified	Intestinal diseases, helminths, anaplasmosis, cowdriosis, babesiosis, sores, retained placenta	(Beinart & Brown, 2013)
Ehretia rigida (Thunb.) Druce	Boraginaceae	Morobe (B)	Roots	Unspecified	Unspecified	Retained placenta	(Van der Merwe et al., 2001)

Plants scientific name	Plant family	#Local name	Part used	Preparation method	Administration mode	Diseases	References
		Unspecified	Roots	Unspecified	Unspecified	Fracture	(Hutchings et al. 1996)
Elephantorrhiza burkei Benth.	Leguminosae	gumululo; tshisesana; tshisesevhafu (V)	Roots /Tubers	Decoction	Unspecified	Anaplasmosis	(Ramovha & van Wyk, 2016)
		Mohauwane (P)	Roots	Unspecified	Unspecified	Babesiosis	(Mongalo & Makhafola, 2018)
		Gumululo (V)	Roots and Bulb	Infusion	Oral	Unspecified	(Luseba & Tshisikhawe, 2013).
Elephantorrhiza elephantina (Burch.) Skeels (*Acacia elephantorrhiza*)	Leguminosae (Fabaceae)	Unspecified	Bulb	Unspecified	Unspecified	Diarrhoea	(Kambizi, 2014)
		iNtololwana (X)	Roots	Decoction	Oral	Cowdriosis	(Dold & Cocks, 2001)
		UHlololwane (X)	Roots	Infusion	Oral	Mange (skin disease)	(Mthi & Rust, 2020)
		Mositsane (S)	Whole plant	Grounding	Unspecified	Retained placenta	(Beinart & Brown, 2013)
		Mositsane (B)	Bulb	Decoction	Unspecified	Diarrhoea, helminths, retained placenta, anaplasmosis, black quarter	(Beinart & Brown, 2013)
		Unspecified	Roots	Maceration	Topical	Anaplasmosis	(Semenya et al., 2019)
		Mohauwane (P)	Roots	Unspecified	Unspecified	Ticks	(Mongalo & Makhafola, 2018)
		Xixuvari (T)	Aerial parts and Bulb	Infusion	Oral	Unspecified	(Luseba & Van der Merwe, 2006).
		Tshisesane (V)	Tubers	Grounding	Topical	Cowdriosis, black quarter	(Magwede et al., 2014)
		Mosetlhane (B)	Rootstock	Unspecified	Unspecified	Wounds	(Van der Merwe et al., 2001)
		Mositsane (B)	Roots	Poultice	Topical	Diarrhoea, coughing,	(Moichwanetse et al, 2020)

Plants scientific name	Plant family	#Local name	Part used	Preparation method	Administration mode	Diseases	References
						pneumonia, cowdriosis	
		Unspecified	Roots	Unspecified	Unspecified	Retained placenta, helminths	(Watt and Breyer-Brandwijk 1962)
Elephantorrhiza obliqua Burtt Davy	Leguminosae	Xixengani (T)	Roots	Decoction	Oral	Diarrhoea, dysentery	(Khunoana et al., 2019)
Englerophytum magalismontanum (Sond.) T.D.Penn. (Syn: *Englerophytum magalismontanum* Krause)	Sapotaceae	Unspecified	Bark	Unspecified	Unspecified	Diarrhoea	(Watt and Breyer-Brandwijk 1962)
		Motlatswa (B)	Roots	Unspecified	Unspecified	Anaplasmosis	(Van der Merwe et al., 2001)
Erythrina caffra Thunb.	Leguminosae	Unspecified	Bark	Unspecified	Unspecified	Fertility problems	(Kambizi, 2014)
		Umsintsi (X)	Unspecified	Infusion	Unspecified	Cowdriosis	(Beinart & Brown, 2013)
Erythrina lysiStemon Hutch.	Leguminosae	Muvhale (V)	Bark	Grounding	Topical	Retained placenta	(Magwede et al., 2014)
Erythrophleum lasianthum Corbishley	Leguminosae	Umbhemise (Z)	Bark	Unspecified	Unspecified	Wounds	(Hutchings et al. 1996)
Euclea undulata Thunb.	Ebenaceae	Umgwali (X)	Leaves	Decoction	Oral	Lung sickness	(Mthi et al. 2020)
Eucomis autumnalis (Mill.) Chitt. (Syn: *Eucomis undulata* Aiton)	Asparagaceae	Unspecified	Unspecified	Unspecified	Unspecified	Cowdriosis	(Masika et al. 1997)
		Ubuhlungu (X)	Leaves	Decoction	Unspecified	Babesiosis and anaplasmosis	(Masika & Afolayan, 2003)
Eucomis bicolor Baker	Asparagaceae	Kgapumpu (S)	Leaves	Infusion	Unspecified	Anaplasmosis	(Beinart & Brown, 2013)
Euphorbia cupularis Boiss. (Syn: *Synadenium cupulare* (Boiss.) L.C.Wheeler ex A.C.White, R.A.Dyer & B.Sloane)	Euphorbiaceae	Mokhoto/Nkonde (T)	Aerial parts	Infusion	Oral	Fertility problems	(Luseba & Van der Merwe, 2006).
		Muswoswo (V)	Stem/ Branch latex	Poultice	Topical	Eye infection, black quarter	(Luseba & Tshisikhawe, 2013).
		Muswoswo (V)	Latex	Maceration	Topical	Eye problems (infections), black quarter, lymph skin disease	(Mabogo, 1990)
		Muswoswo (V)	Leaves and Stem latex	Maceration	Topical	Black quarter, eye problem, fracture	(Magwede et al., 2014)

Plants scientific name	Plant family	#Local name	Part used	Preparation method	Administration mode	Diseases	References
Euphorbia sp.	Euphorbiaceae	Unspecified	Leaves	Unspecified	Unspecified	Wounds	(Kambizi, 2014)
		Unspecified	Stem	Grounding	Unspecified	Skin problems	(Matlebyane et al., 2010).
Euphorbia umbellata (Pax) Bruyns (Syn: *Synadenium grantii* Hook.f.)	Euphorbiaceae	Mdleve(T)	Stem, latex	Maceration	Topical	Retained placenta	(Luseba & Van der Merwe, 2006).
		Mdleve(T)	Stem sap	Maceration	Topical	Eye problems and black quarter	(Khunoana et al., 2019)
Euphorbia cooperi N.E.Br. ex A.Berger	Euphorbiaceae	Mdleve (T)	Stem sap	Maceration	Topical	Eye problems	(Luseba & Van der Merwe, 2006).
Exomis microphylla (Thunb.) Aellen	Amaranthaceae	uMvenyathi (X)	Leaves	Decoction	Oral	Black quarter	(Dold & Cocks, 2001)
Ficus sur Forssk.	Moraceae	UmKhiwane (X)	Leaves	Infusion	Oral	Endometritis, vaginitis	(Mthi & Rust, 2020)
		Unspecified	Bark	Unspecified	Unspecified	Retained placenta	(Kambizi, 2014)
		UmKhiwane (X)	Leaves	Decoction	Unspecified	Wounds	(Masika & Afolayan, 2003)
		Unspecified	Roots	Decoction	Unspecified	Babesiosis, retained placenta	(Watt and Breyer-Brandwijk 1962)
Ficus thonningii Blume	Moraceae	Unspecified	Leaves	Maceration	Topical	Retained placenta	(Semenya et al., 2019)
Garcinia livingstonei T.Anderson	Clusiaceae	Mupimbi (V)	Leaves	Maceration	Unspecified	Wounds	(Luseba & Tshisikhawe, 2013).
Gardenia jasminoides J.Ellis	Rubiaceae	Umtfuma/Itfuma (SS)	Fruits	Decoction	Oral	Eye problems	(Shiba, 2018)
Gardenia volkensii K.Schum.	Rubiaceae	Moralla (B)	Bark	Burn	Unspecified	Helminths	(Beinart & Brown, 2013)
Gasteria croucheri (Hook.f.) Baker	Xanthorrhoeaceae	Ulwimi lenkomo (X)	Unspecified	Infusion	Unspecified	Anthrax	(Beinart & Brown, 2013)
Thesium spp	Santalaceae	Motlhogapele (B)	Whole plant	Decoction	Oral	Babesiosis	(Ndou, 2018)
Gladiolus dalenii Van Geel	Iridaceae	Phende-phende (V)	Bulb and Leaves	Maceration	Topical	Fracture	(Luseba & Tshisikhawe, 2013).
Gloriosa superba L.	Colchicaceae	Ihlamvu (Z)	Unspecified	Unspecified	Unspecified	Eye problems	(Hutchings, 1996)

Plants scientific name	Plant family	#Local name	Part used	Preparation method	Administration mode	Diseases	References
		Unspecified	Corms	Unspecified	Topical	Unspecified	(Gerstner 1939)
		Unspecified	Corms	Unspecified	Topical	Skin eruptions, tick infections, screwworm	(Roberts 1990)
Gnidia capitata L.f.	Thymelaeaceae	iSidikili (X)	Roots	Decoction	Oral	Skin eruptions, tick infections, screwworm	(Dold & Cocks, 2001)
Gomphocarpus fruticosus (L.) W.T.Aiton (*Asclepias* L.)	Apocynaceae	Lebejana/ Mothimolo (S)	Whole plant/ Unspecified	Infusion	Unspecified	Cowdriosis	(Beinart & Brown, 2013)
		Luetsane (B)	Roots	Decoction	Oral	Ticks, wounds, anaplasmosis, Constipation	(Moichwanetse et al, 2020)
		Motimola (B)	Whole plant	Maceration	Oral	Retained placenta, pain	(Ndou, 2018)
Gossypium herbaceum L.	Malvaceae	Muluvha (V)	Seeds	Grounding	Topical	Retained placenta	(Chitura et al., 2018)
Grewia flava DC.	Malvaceae	Moretlwa (B)	Twigs	Poultice	Topical	Eye problems	(Beinart & Brown, 2013)
		Morethla (B)	Roots	Decoction	Oral	Black quarter	(Ndou, 2018)
		Moretlwa (B)	Roots	Unspecified	Unspecified	Diarrhoea	(Van der Merwe et al., 2001)
Grewia lasiocarpa E.Mey. ex Harv.	Malvaceae	Mhlolo (X)	Unspecified	Infusion	Unspecified	Fertility problems	(Beinart & Brown, 2013)
Grewia occidentalis L.	Malvaceae	Umnqabasa (X)	Leaves Twigs	Infusion	Topical	Retained placenta	(Soyelu& Masika, 2009)
		uMnqabaza (X)	Leaves	Infusion	Oral	wounds	(Dold & Cocks, 2001)
		Umnqabaza (X)	Leaves	Decoction	Oral	Anaplasmosis	(Mthi et al. 2020)
Gunnera perpensa L.	Gunneraceae	Unspecified	Rhizomes	Unspecified	Unspecified	Cowdriosis	(Kambizi, 2014)
		Qobo (S)	Bulb	Decoction	Unspecified	Anaplasmosis, urinary infections	(Beinart & Brown, 2013)

Plants scientific name	Plant family	#Local name	Part used	Preparation method	Administration mode	Diseases	References
		Unspecified	Roots	Unspecified	Unspecified	Retained placenta	(Gerstner, 1939)
Gymnanthemum coloratum (Willd.) H.Rob. & B.Kahn (Syn: *Vernonia colorata* (Willd.) Drake)	Compositae	Lowveld tree Vernonia (Eng.)	Roots	Decoction / Infusion	Oral	Retained placenta	(Luseba & Tshisikhawe, 2013).
		Phethane (V)	Leaves	Maceration	Topical	Diarrhoea, Helminths	(Magwede et al., 2014)
Gymnanthemum corymbosum (Thunb.) H.Rob. (Syn: *Vernonia corymbosa* (L.f.) Less., *Vernonia neocorymbosa* Hilliard)	Compositae	Phathaphathane (V)	Roots	Infusion	Oral	Wounds	(Luseba & Tshisikhawe, 2013).
		Unspecified	Roots	Unspecified	Unspecified	Helminths	(Gerstner, 1939)
		Unspecified	Leaves/ Roots	Infusion	Unspecified	Unspecified	Mabogo, 1990
Gymnanthemum mespilifolium (Less.) H.Rob. (Syn: *Vernonia mespilifolia* Less.)	Compositae	Hlungulwana (X)	Stem	Infusion	Oral	Helminths	(Mthi et al. 2020)
Gymnosporia heterophylla (Eckl. & Zeyh.) Loes. (Syn: *Maytenus heterophylla* (Eckl. & Zeyh.) N.Robson)	Celastraceae	Unspecified	Bark & Leaves	Infusion	Unspecified	Babesiosis	(Watt and Breyer-Brandwijk 1962)
Gymnosporia senegalensis (Lam.) Loes	Celastraceae	Xihlangwa (T)	Roots skin	Infusion	Oral	Diarrhoea	(Khunoana et al., 2019)
		Mophato (P)	Roots	Decoction	Oral	Black quarter, diarrhoea	(Mogale, 2017).
		mophato (P)	Unspecified	Unspecified	Unspecified	Diarrhoea	(Matlebyane et al., 2010).
Haemanthus albiflos Jacq.	Amaryllidaceae	uMathunga (X)	Bulb	Grounding	Topical	Diarrhoea, bloat	(Dold & Cocks, 2001)
		Mathunga (X)	Unspecified	Infusion	Unspecified	Fracture	(Beinart & Brown, 2013)
Haplocarpha scaposa Harv.	Compositae	Isikhali (X)	Roots	Decoction	Topical	Anaplasmosis	(Soyelu& Masika, 2009)
Harpagophytum procumbens (Burch.) DC. ex Meisn.	Pedaliaceae	Lematla/Sengaparile (B)	Fruits	Unspecified	Unspecified	Wounds	(Van der Merwe et al., 2001)
		Ntjolovoti (T)	Roots	Infusion	Oral	Retained placenta	(Khunoana et al., 2019)
Harpephyllum caffrum Bernh.	Anacardiaceae	Unspecified	Bark	Unspecified	Unspecified	Diarrhoea, black quarter	(Kambizi, 2014)
Helichrysum caespititium (DC.) Sond. ex Harv.	Compositae	Phate ya ngaka (S)	Leaves	Decoction	Unspecified	Wounds	(Beinart & Brown, 2013)

Plants scientific name	Plant family	#Local name	Part used	Preparation method	Administration mode	Diseases	References
		Phateyangaka (B)	Roots	Infusion / Decoction	Oral	Black quarter, constipation	(Ndou, 2018)
Helichrysum appendiculatum (L.f.) Less.	Compositae	IMpepho (X)	Leaves	Decoction	Unspecified	Coughs, Pains (sores, fractures), diarrhoea	(Masika & Afolayan, 2003)
Helichrysum kraussii Sch.Bip.	Compositae	Tshitambatshedzi (V)	Leaves	Infusion	Topical	Foot rot	(Magwede et al., 2014)
Helichrysum milliganii Hook.f.	Compositae	Muhlomantsetse (SS)	Leaves, stem	Infusion	Oral	Wounds	(Shiba, 2018)
Heteromorpha trifoliata (H.L.Wendl.) Eckl. & Zeyh. (Syn: *Heteromorpha trifoliata* abyssinica)	Apiaceae	Unspecified	Unspecified	Unspecified	Unspecified	Helminths	(Masika et al., 1997)
		UmBangandlela (X)	Roots	Infusion	Oral	Babesiosis, anaplasmosis	(Masika et al., 2000)
Heteromorpha arborescens (Spreng.) Cham. & Schltdl.	Apiaceae	UMbangandlela (X)	Roots	Infusion	Unspecified	Babesiosis, anaplasmosis	(Masika & Afolayan, 2003)
		Muṱhaṱhavhanna (V)	Roots	Infusion	Unspecified	Babesiosis, anaplasmosis	(Ramovha & van Wyk, 2016)
Heteropyxis natalensis Harv.	Myrtaceae	Unspecified	Unspecified	Unspecified	Unspecified	Babesiosis	(Watt and Breyer-Brandwijk 1962)
Hibiscus malacospermus (Turcz.) E.Mey. Ex Harv. (Syn: *Hibiscus malacospermus* E. Meyer)	Malvaceae	UmSongelwa (X)	Roots	Decoction	Oral	Unspecified	(Masika et al., 2000)
Hibiscus diversifolius Jacq.	Malvaceae	UmSongelwa (X)	Roots	Decoction	Unspecified	Retained placenta	(Masika & Afolayan, 2003)
Hippobromus pauciflorus Radlk.	Sapindaceae	Ulathile (uLwathile) (X)	Leaves	Decoction	Topical	Cowdriosis, helminths, retained placenta	(Masika et al., 2000)
		ULathile (X)	Leaves	Decoction	Unspecified	Eye inflammation	(Masika & Afolayan, 2003)
		Ulatile (X)	Leaves	Infusion	Topical	Conjunctivitis	(Soyelu& Masika, 2009)
		iLathile (X)	Bark	Decoction	Oral	wounds	(Dold & Cocks, 2001)
		Unspecified	Roots	Infusion	Unspecified	Cowdriosis, diarrhoea	(Hutchings et al. 1996)

Plants scientific name	Plant family	#Local name	Part used	Preparation method	Administration mode	Diseases	References
		Unspecified	Leaf sap	Unspecified	Unspecified	Cough	(Watt and Breyer-Brandwijk 1962)
Holarrhena pubescens Wall. ex G.Don	Apocynaceae	Mukhaṱhakhaṱ hana (V)	Roots	Decoction /Infusion	Unspecified	Eye inflammation	(Ramovha & van Wyk, 2016)
Hydnora abyssinica A.Br. (Syn: *Hydnora johannis* Becc.)	Hydnoraceae	Lethole (S)	Whole plant	Grounding	Unspecified	Babesiosis	(Beinart & Brown, 2013)
Hyperacanthus amoenus (Sims) Bridson	Rubiaceae	Murombe (V)	Roots	Grounding	Topical	Reproductive diseases	(Luseba & Tshisikhawe, 2013).
		Murombe (V)	Bark	Maceration	Unspecified	Eye problem	(Mahlo, 2006)
Hypoxis colchicifolia Baker	Hypoxidaceae	iNongwe ILabatheki (X)	Corms, Leaves	Decoction	Unspecified	Pain	(Masika & Afolayan, 2003)
		iNongwe ILabatheki (X)	Unspecified	Unspecified	Unspecified	Anaplasmosis, babesiosis	(Masika et al., 1997)
Hypoxis hemerocallidea Fisch., C.A.Mey. & Avé-Lall.	Hypoxidaceae	Maledu/Tshuk u ya poo (B)	Bulb	Poultice	Topical /Oral	Babesiosis and anaplasmosis	(Moichwanetse et al, 2020)
		Maledu/Tshuk u ya poo (B)	Corms	Unspecified	Unspecified	Retained placenta, anaemia, heart problem	(Van der Merwe et al., 2001)
		Monna Maledu (B)	Whole plant	Burn	Topical	Fertility problems, cowdriosis, abortion	(Beinart & Brown, 2013)
Hypoxis rigidula Baker	Hypoxidaceae	Unspecified	Corms	Unspecified	Unspecified	Sores, Diarrhoea	(Kambizi, 2014)
Indigofera cryptantha Harv. (Syn: *Indigofera cryptantha* Benth. ex Harv. var. *cryptantha*)	Leguminosae	kofi (B)	Roots	Decoction	Oral	Black water	(Ndou, 2018)
Indigofera frutescens L.f.	Leguminosae	Unspecified	Roots, bark	Decoction	Unspecified	Diarrhoea	(Watt and Breyer-Brandwijk 1962)
Indigofera sessilifolia DC.	Leguminosae	iKhubalo (X)	Roots	Unspecified	Unspecified	Roundworms	(Dold & Cocks, 2001)
Ipomoea lacunosa L.	Convolvulaceae	Injalamba (X)	Stem	Infusion	Unspecified	Diarrhoea	(Beinart & Brown, 2013)
Jatropha curcas L.	Euphorbiaceae	Nhlampfura (T)	Seeds	Infusion	Oral	Anaplasmosis	(Luseba & Van der Merwe, 2006).

Plants scientific name	Plant family	#Local name	Part used	Preparation method	Administration mode	Diseases	References
		Mupfure (V)	Twigs, leaf latex	Maceration	Topical	Constipation	(Magwede et al., 2014)
		Unspecified	Roots	Infusion	Oral	Wounds	(Semenya et al., 2019)
Jatropha latifolia Pax	Euphorbiaceae	tshipfure (V)	Roots, tubers	Decoction	Unspecified	Helminths	(Ramovha & van Wyk, 2016)
Jatropha zeyheri Sond.	Euphorbiaceae	Seswagadi (B)	Roots	Decoction	Topical	Babesiosis	(Beinart & Brown, 2013)
		Xidomeja/ Mudomeja (T)	Roots	Infusion	Oral	Retained placenta	(Luseba & Van der Merwe, 2006).
		Seswagadi (B)	Roots	Poultice	Topical	Ill-thrift, diarrhoea	(Moichwanetse et al, 2020)
		Sefapabadia (P)	Roots	Unspecified	Unspecified	Retained placenta, kidney stone	(Mongalo & Makhafola, 2018)
Kedrostis africana (L.) Cogn.	Cucurbitaceae	UTuvana (X)	Bark, leaves	Decoction	Unspecified	Unspecified	(Masika & Afolayan, 2003)
		uTuvana (X)	Unspecified	Unspecified	Unspecified	Anaplasmosis	(Masika et al., 1997)
Kiggelaria africana L.	Achariaceae	Lekatse (S)	Roots	Infusion	Unspecified	Babesiosis, anaplasmosis	(Beinart & Brown, 2013)
Kleinia longiflora DC.	Compositae	Mosiama (B)	Whole plant	Unspecified	Topical	Retained placenta	(Ndou, 2018)
Lantana camara L.	Verbenaceae	Unspecified	Leaves	Infusion	Topical	Fracture	(Moyo,2008)
		Unspecified	Leaves	Maceration	Topical	Ticks	(Semenya et al., 2019)
Lantana rugosa Thunb.	Verbenaceae	Unspecified	Leaves	Unspecified	Unspecified	Ticks	(Watt and Breyer-Brandwijk 1962)
Ledebouria cooperi (Hook.f.) Jessop	Asparagaceae	Icubudwana (Z)	Unspecified	Unspecified	Unspecified	Eye problems	(Hutchings, 1996)
		Unspecified	Unspecified	Unspecified	Unspecified	Unspecified	(Watt and Breyer-Brandwijk 1962)
Ledebouria revoluta (L.f.) Jessop	Asparagaceae	Inqwebebane (X)	Leaves	Decoction	Unspecified	Reproductive diseases	(Masika & Afolayan, 2003)
		Unspecified	Bulb	Unspecified	Unspecified	Anaplasmosis	(Watt and Breyer-Brandwijk 1962)
Leonotis leonurus (L.) R.Br.	Lamiaceae	Imunyamunya (X)	Roots, leaves	Infusion	Oral	Anaplasmosis	(Hutchings, 1996)

Plants scientific name	Plant family	#Local name	Part used	Preparation method	Administration mode	Diseases	References
		UmFincafincane (X)	Leaves	Maceration	Topical	Anaplasmosis	(Masika et al., 2000)
		Unspecified	Roots, leaves	Maceration	Unspecified	Eye inflammation	(Hulme 1954)
		UmFincafincane (X)	Leaves	Maceration	Unspecified	Helminths	(Masika & Afolayan, 2003)
Leonotis ocymifolia (Burm.f.) Iwarsson	Lamiaceae	Umunyane (Z)	Roots, leaves	Infusion	Oral	Conjunctivitis	(Hutchings, 1996)
		Unspecified	Roots, leaves	Maceration	Unspecified	Anaplasmosis	(Hulme 1954)
Leucas capensis (Benth.) Engl.	Lamiaceae	uPhiphiyo (X)	Leaves	Decoction	Oral	Helminths	(Dold & Cocks, 2001)
Lippia javanica (Burm.f.) Spreng.	Verbenaceae	Unspecified	Leaves	Maceration	Topical	Anaplasmosis	(Semenya et al., 2019)
		Mosinkwane (P)	Unspecified	Unspecified	Unspecified	Wounds	(Matlebyane et al., 2010).
		InZinziniba (X)	Leaves	Decoction	Unspecified	Insect repellent	(Masika & Afolayan, 2003)
Macrotyloma axillare (E.Mey.) Verdc.	Leguminosae	Unspecified	Leaves, stem	Infusion	Unspecified	Anaplasmosis, babesiosis	(Hulme 1954)
Maerua angolensis DC.	Capparaceae	Mutambanamme (V)	Leaves	Infusion	Oral	Swollen udder	(Luseba & Tshisikhawe, 2013).
Marrubium vulgare L.	Lamiaceae	uMhlonyane (X)	Leaves	Decoction	Oral	Anaplasmosis	(Dold & Cocks, 2001)
Maytenus peduncularis Loes. (Syn: *Maytenus eduncularis*)	Celastraceae	Mukwatukwatu (V)	Root-bark	Poultice	Topical	Fractures	(Chitura et al., 2018)
Melia azedarach L.	Meliaceae	UmSeringa (X)	Leaves	Decoction	Unspecified	Anaplasmosis	(Masika & Afolayan, 2003)
		umSeringa (X)	Unspecified	Unspecified	Unspecified	Babesiosis and anaplasmosis	(Masika et al., 1997)
		Unspecified	Leaves	Decoction	Topical	Conjunctivitis	(Semenya et al., 2019)
Merwilla plumbea (Lindl.) Speta (Syn: *Scilla natalensis* Planch.)	Asparagaceae	Rramburo (S)	Bulb	Infusion	Unspecified	Retained placenta	(Beinart & Brown, 2013)
		ichitha (Z)	Unspecified	Unspecified	Unspecified	Lung sickness	(Hutchings et al. 1996)
Monsonia emarginata L'Hér.	Geraniaceae	Unspecified	Unspecified	Unspecified	Unspecified	Stomach problem	(Watt and Breyer-Brandwijk 1962)

Plants scientific name	Plant family	#Local name	Part used	Preparation method	Administration mode	Diseases	References
Nicotiana tabacum L.	Solanaceae	Unspecified	Leaves	Decoction & Maceration	Oral/Topical	Constipation, conjunctivitis, wounds	(Semenya et al., 2019)
		Motsoko (B)	Leaves	Unspecified	Unspecified	Conjunctivitis	(Van der Merwe et al., 2001)
Ochna holstii Engl.	Ochnaceae	Tshipfure (V)	Leaves	Decoction	Topical	Wounds	(Magwede et al., 2014)
Olea europaea L.	Oleaceae	MNquma (X)	Bark	Decoction	Unspecified	Anaplasmosis	(Masika & Afolayan, 2003)
		mNquma (X)	Unspecified	Unspecified	Unspecified	Babesiosis and anaplasmosis	(Masika et al., 1997)
		uMnquma (X)	Leaves	Unspecified	Unspecified	Endometritis, vaginitis, anaplasmosis	(Dold & Cocks, 2001)
		Umkhondo (X)	Bark	Infusion	Oral	Black quarter	(Mthi et al., 2018)
		Unspecified	Leaves	Infusion	Oral	Constipation	(Semenya et al., 2019)
Opuntia ficus-indica (L.) Mill.	Cactaceae	Toorofeye (B)	Flower	Poultice	Topical	Retained placenta	(Moichwanetse et al, 2020)
Osyris lanceolata Hochst. & Steud.	Santalaceae	Mpera (B)	Bulb	Maceration	Oral	Retained placenta, pain, internal bleeding	(Moichwanetse et al, 2020)
Ozoroa paniculosa (Sond.) R.Fern. & A.Fern.	Anacardiaceae	Monokane (B)	Bark, root-bark	Unspecified	Unspecified	Babesiosis, diarrhoea, wweating sickness	(Van der Merwe et al., 2001)
		Unspecified	Bark	Unspecified	Unspecified	Stomach problems	(Hutchings et al. 1996);
Pappea capensis Eckl. & Zeyh.	Sapindaceae	Unspecified	Bark	Unspecified	Unspecified	Helminths	(Mphahlele, 2016)
		Imfuce (SS)	Bark	Infusion	Oral	Helminths	(Shiba, 2018)
		Unspecified	Unspecified	Unspecified	Unspecified	Unspecified	(Gerstner, 1939)
Pavetta revoluta Hochst.	Rubiaceae	USkolpati (X)	Leaves	Infusion	Unspecified	Anaplasmosis	(Masika & Afolayan, 2003)
Pelargonium caffrum (Eckl. & Zeyh.) Steud.	Geraniaceae	Kgware (B)	Bulb	Decoction	Unspecified	Anaplasmosis	(Beinart & Brown, 2013)
		Unspecified	Leaves	Infusion	Unspecified	Unspecified	(Hutchings et al. 1996)

Plants scientific name	Plant family	#Local name	Part used	Preparation method	Administration mode	Diseases	References
Pelargonium luridum (Andrews) Sweet	Geraniaceae	Unspecified	Leaves	Infusion	Unspecified	Unspecified	(Hutchings et al. 1996)
Pelargonium reniforme Curtis	Geraniaceae	Uvendle (X)	Unspecified	Unspecified	Unspecified	Anaplasmosis	(Beinart & Brown, 2013)
		iNtololwana, uVendle (X)	Roots	Infusion	Oral	Diarrhoea, cowdriosis	(Dold & Cocks, 2001)
		iVendle Kubalo (X)	Roots	Decoction	Unspecified	Anaplasmosis	(Masika & Afolayan, 2003)
Pelargonium sidoides DC.	Geraniaceae	Unspecified	Unspecified	Decoction	Unspecified	Helminths	(Watt and Breyer-Brandwijk 1962)
Peltophorum africanum Sond.	Leguminosae	Unspecified	Bark	Infusion	Oral	Intestine parasites	(Semenya et al., 2019)
		Mosehla (P)	Leaves	Unspecified	Oral	Unspecified	(Mongalo & Makhafola, 2018)
		Musese (V)	Bark	Grounding	Topical	Wounds	(Magwede et al., 2014)
		Unspecified	Bark	Unspecified	Unspecified	Helminths	(Mphahlele, 2016)
		Mosetla (B)	Root-bark, bark	Unspecified	Unspecified	Diarrhoea	(Van der Merwe et al., 2001)
		Mosetlha (B)	Leaves, bark	Poultice	Topical/Oral	Retained placenta, Diarrhoea, blood clots	(Moichwanetse et al, 2020)
Pentanisia prunelloides (Klotzsch) Walp.	Rubiaceae	Icimamlilo (X)	Leaves	Decoction	Unspecified	Conjunctivitis, retained placenta	(Masika & Afolayan, 2003)
		Unspecified	Roots	Unspecified	Unspecified	Skin problems, anaplasmosis	(Kambizi, 2014)
		Unspecified	Roots	Decoction	Unspecified	Retained placenta	(Watt and Breyer-Brandwijk 1962)
Persea americana Mill.	Lauraceae	Unspecified	Roots	Maceration	Topical	Wounds	(Semenya et al., 2019)
Philenoptera violacea (Klotzsch) Schrire	Leguminosae	Mufhanda (V)	Bark	Decoction	Topical	Wounds	(Magwede et al., 2014)
		mufhanda (V)	Stem/root-bark	Decoction /Infusion	Unspecified	Babesiosis	(Ramovha & van Wyk, 2016)
		Mbhandzu (T)	Bark	Infusion	Oral	Gall, diarrhoea	(Khunoana et al., 2019)
Phyllanthus parvulus Sond. (Syn: *Phyllanthus burchellii* Müll.Arg.)	Phyllanthaceae	Lentsane (B)	Aerial parts	Unspecified	Unspecified	Conjunctivitis	(Van der Merwe et al., 2001)

Plants scientific name	Plant family	#Local name	Part used	Preparation method	Administration mode	Diseases	References
Phytolacca heptandra Retz.	Phytolaccaceae	Umnyanja (X)	Leaves	Infusion /Decoction	Topical	Wounds	(Soyelu& Masika, 2009)
		Unspecified	Roots	Unspecified	Unspecified	Lung sickness	(Watt and Breyer-Brandwijk 1962)
Phytolacca octandra L.	Phytolaccaceae	Unspecified	Roots	Infusion	Unspecified	Lung sickness	(Watt and Breyer-Brandwijk 1962)
Piper nigrum L.	Piperaceae	Unspecified	Seeds	Maceration/ Infusion	Topical/ Oral	Wounds, helminths	(Semenya et al., 2019)
Pittosporum viridiflorum Sims	Pittosporaceae	Umkhwenkhwe (X)	Bark	Infusion	Unspecified	Anaplasmosis	(Beinart & Brown, 2013)
		Umkwenkwe (X)	Roots	Decoction	Topical	Wounds	(Soyelu& Masika, 2009)
		UmKwenkwe (X)	Bark	Decoction and Infusion	Unspecified	Anaplasmosis	(Masika & Afolayan, 2003)
		UmKwenkwe (X)	Bark	Decoction	Oral	Anaplasmosis	(Masika et al., 2000)
Plectranthus ambiguus (Bolus) Codd	Lamiaceae	Irhajojo (X)	Leaves and Stem	Infusion and Maceration	Topical	Wounds, maggot-infested wounds	(Soyelu& Masika, 2009)
Plectranthus laxiflorus Benth.	Lamiaceae	Unspecified	Unspecified	Unspecified	Unspecified	Unspecified	(Watt and Breyer-Brandwijk 1962)
		UHlololwane (X)	Roots, bark	Decoction	Unspecified	Anaplasmosis, babesiosis, retained placenta	(Masika & Afolayan, 2003)
		uHlololwane (X)	Unspecified	Unspecified	Unspecified	Babesiosis and anaplasmosis	(Masika et al., 1997)
Plumbago auriculata Lam.	Plumbaginaceae	uTshintshini (X)	Roots	Infusion	Oral	Diarrhoea	(Dold & Cocks, 2001)
Plumbago zeylanica L.	Plumbaginaceae	Masegomabe (B)	Roots	Unspecified	Unspecified	Pneumonia	(Van der Merwe et al., 2001)
Podocarpus latifolius (Thunb.) R.Br. ex Mirb.	Podocarpaceae	umKoba UmKoba (X)	Bark	Decoction	Unspecified	Anaplasmosis	(Masika & Afolayan, 2003)
		umKoba UmKoba (X)	Bark	Decoction	Oral	Anaplasmosis	(Masika et al., 2000)

Plants scientific name	Plant family	#Local name	Part used	Preparation method	Administration mode	Diseases	References
Pouzolzia mixta Solms	Urticaceae	Mongololo or mongollo (B)	Roots	Infusion	Unspecified	Retained placenta, dystocia, anaplasmosis, diarrhoea	(Beinart & Brown, 2013)
		Mongololo (B)	Roots	Poultice	Topical	Retained placenta	(Moichwanetse et al, 2020)
		Mongololo (B)	Roots, leaves	Unspecified	Unspecified	Retained placenta, bloat, vaginal discharge	(Van der Merwe et al., 2001)
Printzia pyrifolia Less.	Compositae	Unspecified	Roots	Unspecified	Unspecified	Unspecified	((Gerstner, 1939))
Protea caffra Meisn.	Proteaceae	Isiqwani (X)	Seeds	Decoction	Oral	Cowdriosis	(Mthi et al. 2020)
		Unspecified	Unspecified	Unspecified	Unspecified	Diarrhoea	(Hutchings et al. 1996)
Protea welwitschii Engl.	Proteaceae	Unspecified	Roots	Infusion	Unspecified	Dysentery, diarrhoea	(Watt and Breyer-Brandwijk 1962)
Protorhus longifolia (Bernh.) Engl.	Anacardiaceae	uZintlwa (X)	Bark	Decoction	Oral	Cowdriosis, diarrhoea	(Dold & Cocks, 2001)
Prunus persica (L.) Batsch	Rosaceae	Impitchi (X)	Unspecified	Poultice	Unspecified	Tick wounds, myiasis	(Beinart & Brown, 2013)
		Muberegisi (V)	Leaves	Grounding	Topical	Wounds, eye problems	(Luseba & Tshisikhawe, 2013).
		Ipesika (X)	Leaves	Infusion	Topical	Wounds	(Soyelu& Masika, 2009)
		Muberegisi (V)	Leaves	Grounding	Topical	Wounds	(Magwede et al., 2014)
Ptaeroxylon obliquum (Thunb.) Radlk.	Rutaceae	Umbhaqa (Z)	wood	Unspecified	Unspecified	Anthrax, Ticks	(Hutchings, 1996)
		Umthathi (X)	Unspecified	Decoction	Unspecified	Anaplasmosis, bovine ephemeral fever	(Beinart & Brown, 2013)
		Umthathi (X)	Leaves, bark	Infusion / Maceration	Topical	Wounds	(Soyelu& Masika, 2009)
		uBhaqa (X)	Bark	Infusion	Topical	Ticks	(Moyo,2008)

Plants scientific name	Plant family	#Local name	Part used	Preparation method	Administration mode	Diseases	References
Pterocarpus angolensis DC.	Leguminosae	Vhangazi/ Murhotso (T)	Bark	Infusion	Oral	Unthriftiness, anaplasmosis, helminths, black quarter	(Luseba & Van der Merwe, 2006).
		Mutondo (V)	Bark	Infusion	Oral	Babesiosis	(Luseba & Tshisikhawe, 2013).
		Mutondo (V)	Bark	Grounding	Topical	Wounds	(Magwede et al., 2014)
		Mutondo (V)	Stem/root-bark	Decoction /Infusion	Unspecified	Babesiosis	(Ramovha & van Wyk, 2016)
		Mutondo (V)	Stem-bark	Infusion	Oral	Constipation	(Chitura et al., 2018)
Quercus robur L.	Fagaceae	UmOkhi (X)	Bark	Decoction	Unspecified	Anaplasmosis	(Masika & Afolayan, 2003)
Rapanea melanophloeos (L.) Mez	Primulaceae	Chithibunga (X)	Bulb	Decoction	Oral	Cowdriosis	(Dold & Cocks, 2001)
Rauvolfia caffra Sond.	Apocynaceae	Munadzi (V)	Bark	Grounding	Topical	Wounds	(Magwede et al., 2014)
Rhamnus prinoides L'Hér.	Rhamnaceae	Mofifi (S)	Leaves	Decoction	Topical	Anaplasmosis, Ticks, foot rot	(Beinart & Brown, 2013)
Rhoicissus digitata (L. f.) Gilg & M. Brandt	Vitaceae	Isinwazi (X)	Tubers	Unspecified	Unspecified	Unspecified	(Hutchings, 1996)
Rhoicissus tomentosa (Lam.) Wild & R.B.Drumm.	Vitaceae	uChithibhunga (X)	Bark	Decoction	Oral	Retained placenta	(Mthi et al. 2020)
		uChithibhunga (X)	Bulb	Decoction	Oral	Babesiosis	(Mthi et al. 2020)
		iSaqoni Sakoone (X)	Bark, roots	Decoction	Unspecified	Cowdriosis, bovine ephemeral fever, Helminths	(Masika & Afolayan, 2003)
		Isaqoni (X)	Bark	Decoction	Oral	Bovine ephemeral fever	(Masika et al., 2000)
		Unspecified	Roots	Unspecified	Unspecified	Helminths	(Watt and Breyer-Brandwijk 1962)
Rhoicissus tridentata (L.f.) Wild & R.B.Drumm.	Vitaceae	Isinwazi (Z)	Tubers	Unspecified	Unspecified	Unspecified	(Hutchings, 1996)
		Ntagaraga (B)	Tubers	Unspecified	Unspecified	Babesiosis, helminths,	(Van der Merwe et al., 2001)

Plants scientific name	Plant family	#Local name	Part used	Preparation method	Administration mode	Diseases	References
						abortion, cowdriosis	
		Unspecified	Tubers	Unspecified	Unspecified	Unspecified	(Pujol 1990)
Rhus incisa L.f.	Anacardiaceae	uNongquthu (X)	Roots, bark	Infusion	Oral	Shock, diarrhoea	(Dold & Cocks, 2001)
Rhynchosia komatiensis Harms	Leguminosae	murudamali (V)	Roots	Decoction	Unspecified	Babesiosis	(Ramovha & van Wyk, 2016)
Ricinus communis L.	Euphorbiaceae	Mokhura (B)	Seeds	Unspecified	Unspecified	Helminths, constipation	(Van der Merwe et al., 2001)
		Unspecified	Seeds	Maceration	Unspecified	Constipation	(Hutchings et al. 1996)
Rothmannia capensis Thunb.	Rubiaceae	Murathamapfene/Murathambila (V)	Fruits	Grounding	Topical	Wounds	(Magwede et al., 2014)
Rumex acetosa L.	Polygonaceae	Kgamane (S)	Roots	Decoction	Unspecified	Anaplasmosis	(Beinart & Brown, 2013)
Rumex lanceolatus Thunb.	Polygonaceae	IDololenkonyana (X)	Rhizomes	Decoction	Unspecified	Cowdriosis, helminths	(Masika & Afolayan, 2003)
Salix capensis Thunb.	Salicaceae	Umnonono (X)	Bark	Decoction	Oral	Retained placenta	(Mthi & Rust, 2020)
		UmNgcunube (X)	Bark, leaves	Infusion	Oral	Anaplasmosis, babesiosis, retained placenta	(Masika & Afolayan, 2003)
		UmNgcunube (X)	Bark and Leaves	Decoction	Unspecified	Babesiosis and anaplasmosis	(Masika et al., 1997)
Salix mucronata Thunb.	Salicaceae	Unspecified	Unspecified	Unspecified	Unspecified	Retained placenta	(Masika et al., 2000)
		Moduane (S)	Unspecified	Unspecified	Unspecified	Helminths	(Beinart & Brown, 2013)
Salvia runcinata L.f.	Lamiaceae	Mosisidi (S)	Roots	Decoction	Unspecified	Anaplasmosis	(Beinart & Brown, 2013)
Sarcophyte sanguinea Sparrm.	Balanophoraceae	Umnquma (X)	Whole plant	Infusion	Oral	Black quarter	(Mthi et al., 2018)
Schizocarphus nervosus (Burch.) van der Merwe (Syn: *Scilla nervosa* (Burch.) J.P.Jessop)	Asparagaceae	Unspecified	Unspecified	Unspecified	Unspecified	Constipation	(Gerstner 1941)

**Plants scientific name**	**Plant family**	**#Local name**	**Part used**	**Preparation method**	**Administration mode**	**Diseases**	**References**
Schkuhria pinnata (Lam.) Kuntze ex Thell.	Compositae	Santhloko/ lefero (B)	Aerial parts	Unspecified	Unspecified	Pneumonia, diarrhoea, cowdriosis, conjunctivitis	(Van der Merwe et al., 2001)
Schotia brachypetala Sond.	Leguminosae	Unspecified	Leaves	Unspecified	Unspecified	Helminths	(Mphahlele, 2016)
		mulubi (V)	Stem/rootbark	Decoction	Unspecified	Babesiosis	(Ramovha & van Wyk, 2016)
		Chochela-mandleni (T)	Bark	Decoction	Oral	Foot and mouth disease, Black quarter	(Khunoana et al., 2019)
Schotia latifolia Jacq.	Leguminosae	Umgxam (X)	Bark	Decoction	Topical	Wounds	(Soyelu& Masika, 2009)
		UmGxam (X)	Bark, leaves	Decoction	Unspecified	Babesiosis	(Masika & Afolayan, 2003)
		Unspecified	Roots	Unspecified	Unspecified	Skin problems	(Kambizi, 2014)
		uMgxam (X)	Bark	Decoction	Oral	Babesiosis	(Dold & Cocks, 2001)
Sclerocarya birrea (A.Rich.) Hochst.	Anacardiaceae	Mufula (V)	Stembark	Decoction /Infusion	Unspecified	Babesiosis	(Ramovha & van Wyk, 2016)
		Morula (P)	Stembark	Unspecified	Unspecified	Unspecified	(Mongalo & Makhafola, 2018)
		Morula (B)	Bark	Unspecified	Unspecified	Diarrhoea, fracture	(Van der Merwe et al., 2001)
Searsia lancea (L.f.) F.A.Barkley (Syn: *Rhus lancea* L.f.)	Anacardiaceae	Moshabele (B)	Root-bark	Unspecified	Unspecified	Diarrhoea, Anaplasmosis	(Van der Merwe et al., 2001)
		Mushakaladza (V)	Leaves	Decoction	Oral	Lumpy skin disease	(Luseba & Tshisikhawe, 2013).
Searsia pyroides (Burch.) Moffett	Anacardiaceae	Bohitlha (B)	Roots	Poultice	Topical	Retained placenta	(Moichwanetse et al, 2020)
Secamone filiformis J.H. Ross	Apocynaceae	iMbijela (X)	Stem	Infusion	Oral	Diarrhoea	(Dold & Cocks, 2001)
Seddera suffruticosa Hallier f.	Convolvulaceae	Thobeha (B)	Bulb	Unspecified	Topical	Fractures	(Beinart & Brown, 2013)
		Thobega (B)	Roots	Unspecified	Unspecified	Fracture	(Van der Merwe et al., 2001)
Senecio barbertonicus Klatt	Compositae	Intseleti (SS)	Leaves	Infusion	Oral	Helminths	(Shiba, 2018)
Senecio oxyriifolius DC.	Compositae	Unspecified	Leaves	Unspecified	Unspecified	Swellings	(Hutchings et al. 1996)

Plants scientific name	Plant family	#Local name	Part used	Preparation method	Administration mode	Diseases	References
Senecio tamoides DC.	Compositae	ihlozi elikhulu (Z)	Unspecified	Unspecified	Unspecified	Anthrax, black quarter	(Hutchings, 1996)
		Unspecified	Unspecified	Unspecified	Unspecified	Unspecified	(Gerstner, 1939)
Senna sophera (L.) Roxb. (Syn: *Cassia sophera* L.)	Leguminosae	Mutsheketshek e (V)	Aerial parts	Grounding	Topical	Wounds	(Magwede et al., 2014)
Senna italica Mill.	Leguminosae	Sebetebete (B)	Unspecified	Infusion	Unspecified	Anaplasmosis, retained placenta	(Beinart & Brown, 2013)
		Sebete (B)	Roots	Decoction	Unspecified	Constipation, diarrhoea, anaplasmosis, anthrax	(Beinart & Brown, 2013)
		Xintomane (T)	Roots	Decoction	Oral	Unspecified	(Khunoana et al., 2019)
		Ximbangamba ngana (T)	Bark	Infusion	Oral	Diarrhoea, anaplasmosis	(Luseba & Van der Merwe, 2006).
		Unspecified	Root-bark	Unspecified	Unspecified	Helminths	(Mphahlele, 2016)
		Sebetebete (B)	Whole plant	Decoction / Infusion	Oral	Anaplasmosis, diarrhoea, gastrointestina l parasites, pains (from sores, fractures)	(Ndou, 2018)
		Sebetebete /Sebete/ Monyokololo (B)	Unspecified	Unspecified	Unspecified	Anaplasmosis	(Getchell et al 2001)
		Sebete (B)	Roots	Unspecified	Unspecified	Anaplasmosis, intestinal diseases, cowdriosis, anthrax, pneumonia	(Van der Merwe et al., 2001)

Plants scientific name	Plant family	#Local name	Part used	Preparation method	Administration mode	Diseases	References
		tshiḓuwaḓuwane; muḓuwaḓuwane (V)	Whole plant	Decoction	Unspecified	Babesiosis	(Ramovha & van Wyk, 2016)
		Sebetebete (B)	Roots	Poultice	Topical	Retained placenta	(Moichwanetse et al, 2020)
Senna petersiana (Bolle) Lock	Leguminosae	munembenembe; gonela (V)	Roots	Infusion	Unspecified	Babesiosis	(Ramovha & van Wyk, 2016)
Senna tora (L.) Roxb.	Leguminosae	Mongepenpe (B)	Whole plants	Poultice	Topical	Retained placenta	(Moichwanetse et al, 2020)
Sideroxylon inerme L.	Sapotaceae	uMqwashu (X)	Bark	Decoction	Oral	Babesiosis	(Dold & Cocks, 2001)
Solanum hermannii Dunal (Syn: *Solanum sodomeum* L.)	Solanaceae	Umthuma (X)	Leaves,fruits	Infusion and Maceration	Topical	Wounds	(Soyelu& Masika, 2009)
Solanum aculeastrum Dunal	Solanaceae	intuma, umthuma (Z)	Fruits	Unspecified	Unspecified	Ringworms	(Hutchings, 1996)
		Mututulwa (V)	Fruits	Grounding	Topical	Wounds	(Magwede et al., 2014)
Solanum anguivi Lam.	Solanaceae	Umthuma (X)	Leaves	Decoction	Unspecified	Retained placenta	(Masika & Afolayan, 2003)
Solanum campylacanthum Hochst.	Solanaceae	Tolwane e nyane (B)	Roots	Decoction	Oral	Pain	(Ndou, 2018)
Solanum capense L.	Solanaceae	Unspecified	Fruits	Unspecified	Unspecified	Warts, ringworm	(Watt and Breyer-Brandwijk 1962)
Solanum incanum L.	Solanaceae	Mututulwa muhulwane (V)	Fruits	Grounding	Topical	Eye problems	(Luseba & Tshisikhawe, 2013).
		Tolwana (B)	Roots	Unspecified	Unspecified	Sores	(Van der Merwe et al., 2001)
Solanum lichtensteinii Willd.	Solanaceae	Tolwane (B)	Flower	Poultice	Topical	Diarrhoea, Intestinal parasites, Fracture	(Moichwanetse et al, 2020)
		Tolwane (B)	Roots	Infusion	Oral	Internal sores caused by gastrointestinal parasites	(Ndou, 2018)

Plants scientific name	Plant family	#Local name	Part used	Preparation method	Administration mode	Diseases	References
		Ndhulwani(T)	Aerial parts	Infusion	Oral/topical	Respiratory problems	(Luseba & Van der Merwe, 2006).
Solanum mauritianum Scop.	Solanaceae	uMbangabanga (X)	Roots	Decoction	Oral	Dystocia	(Dold & Cocks, 2001)
Solanum panduriforme E. Mey.	Solanaceae	intuma-omncane (Z)	Fruit sap	Unspecified	Unspecified	Sore eyes, ringworm	(Hutchings, 1996)
		Motholla (P)	Unspecified	Unspecified	Unspecified	upset stomach	(Matlebyane et al., 2010).
		Motholla (P)	Whole plant	Infusion	Oral	Constipation	(Mogale, 2017).
		Mohato (B)	Fruit sap	Unspecified	Unspecified	Diarrhoea	(Van der Merwe et al., 2001)
Solanum supinum Dunal	Solanaceae	Mututuwa (V)	Fruits	Grounding	Topical	Wounds	(Magwede et al., 2014)
Sphedamnocarpus pruriens (A. Juss.) Szyszył.	Malpighiaceae	Makgonatsotlhe (B)	Roots	Infusion	Unspecified	Retained placenta, eye problem, black quarter	(Beinart & Brown, 2013)
Spirostachys africana Sond.	Euphorbiaceae	Injuqu (Z)	Sap & wood	Unspecified	Unspecified	maggot repellent	(Hutchings, 1996)
		Muonze (V)	Bark	Grounding	Topical	Wounds	(Magwede et al., 2014)
		Morekhure (B)	Wood	Unspecified	Unspecified	Sweating sickness	(Van der Merwe et al., 2001)
Stangeria eriopus (Kunze) Baill.	Zamiaceae	Imfingwana (X)	Roots	Infusion	Unspecified	Paratyphoid	(Beinart & Brown, 2013)
		uMfingwani (X)	Root-bark	Grounding	Oral	Helminths	(Dold & Cocks, 2001)
		Unspecified	Roots	Unspecified	Unspecified	Cowdriosis	(Kambizi, 2014)
Streptocarpus prolixus C.B. Clarke	Gesneriaceae	Unspecified	Leaves	Infusion	Unspecified	Constipation	(Hulme 1954)
Strychnos decussata (Pappe) Gilg	Loganiaceae	uMnonono (X)	Bark	Infusion	Oral	Roundworms	(Dold & Cocks, 2001)
Strychnos henningsii Gilg	Loganiaceae	Umnonono (X)	Bark	Infusion	Oral	Babesiosis	(Mthi et al. 2020)
		uMnonono (X)	Bark	Decoction	Oral	Paratyphoid	(Mthi et al., 2018)
		uMfingwani (X)	Root-bark	Grounding	Oral	Cowdriosis, diarrhoea	(Dold & Cocks, 2001)

Plants scientific name	Plant family	#Local name	Part used	Preparation method	Administration mode	Diseases	References
Synadenium cupulare L.C. Wheeler	Euphorbiaceae	Muswoswo (V)	Resin	Unspecified	Topical	Arthritis	(Chitura et al., 2018)
Tabernaemontana elegans Stapf	Apocynaceae	Muhaṱu (V)	Roots	Decoction /Infusion	Unspecified	Babesiosis	(Ramovha & van Wyk, 2016)
Tagetes minuta L.	Compositae	khaki bush (Eng.)	Leaves	Infusion	Topical	Ticks	(Moyo,2008)
		Mushushathuri (V)	Leaves	Grounding	Topical	Ticks	(Luseba & Tshisikhawe, 2013).
		Khaki weed (Eng.)	Leaves	Grounding	Topical	Wounds	(Magwede et al., 2014)
Tapinanthus oleifolius (J.C.Wendl.) Danser	Loranthaceae	Moshitlwane (S)	Roots	Decoction	Topical	Blindness, stiff joints	(Beinart & Brown, 2013)
Tarchonanthus camphoratus L.	Compositae	Moologa (B)	Leaves	Maceration	Oral	Retained placenta, pain alleviation	(Moichwanetse et al, 2020)
		Mohatlha (B)	Leaves	Infusion	Oral	To prevent cold	(Ndou, 2018)
Tephroseris palustris (L.) Rchb. (Syn: *Senecio congestus* (R.Br.) DC.)	Compositae	Lichama (SS)	Roots	Decoction	Oral	helminths	(Shiba, 2018)
Tephrosia kraussiana Meissner	Leguminosae	Insangwana (Z)	Whole plant	Unspecified	Unspecified	Black quarter	(Hutchings, 1996)
		Unspecified	Unspecified	Unspecified	Unspecified	Black quarter	(Doke and Vilakazi 1972)
Tephrosia macropoda (E.Mey.) Harv.	Leguminosae	Unspecified	Roots, seeds	Unspecified	Unspecified	Vermin	(Gerstner 1941)
		Unspecified	Leaves	Unspecified	Unspecified	Helminths	(Bryant 1966)
Terminalia sericea Burch. ex DC.	Combretaceae	Mogonono (B)	Leaves	Poultice	Topical	Retained placenta	(Moichwanetse et al, 2020)
		Mususu (V)	Roots	Decoction / Infusion	Oral/ Topical	Diarrhoea, Ticks, wound	(Luseba & Tshisikhawe, 2013).
		Konono (T)	Leaves	Poultice	Topical	Wound	(Luseba & Van der Merwe, 2006).
		Mususu (V)	Roots	Infusion	Topical	Ticks, wounds	(Magwede et al., 2014)
		Mogonono (B)	Roots	Unspecified	Unspecified	Diarrhoea	(Van der Merwe et al., 2001)

Plants scientific name	Plant family	#Local name	Part used	Preparation method	Administration mode	Diseases	References
		Mususu (V)	Root-bark	Infusion	Unspecified	Babesiosis	(Ramovha & van Wyk, 2016)
Tetradenia riparia (Hochst.) Codd	Lamiaceae	iboza, ibozane (Z)	Leaves	Unspecified	Unspecified	Anaplasmosis, fevers	(Hutchings, 1996)
Teucrium africanum Thunb.	Lamiaceae	uBuhlungu bebhokhwe (X)	Leaves	Unspecified	Unspecified	Anaplasmosis	(Dold & Cocks, 2001)
Teucrium trifidum Retz. (Syn: *Teucrium capense* Thunb.)	Lamiaceae	Unspecified	Unspecified	Unspecified	Unspecified	Babesiosis and anaplasmosis	(Masika et al., 1997)
		ubuhlungu be Bhokwe (X)	Leaves	Decoction	Unspecified	Anaplasmosis	(Masika & Afolayan, 2003)
		Lethe la noga (B)	Whole plant	Infusion	Oral	Reproduction problems	(Ndou, 2018)
Trema orientalis (L.) Blume	Cannabaceae	Mukurukuru (V)	Leaves	Infusion	Oral	Eye problems, anaplasmosis	(Luseba & Tshisikhawe, 2013).
Tribulus terrestris L.	Zygophyllaceae	Unspecified	Leaves	Decoction	Unspecified	Reproduction problems, Diarrhoea	(Semenya et al., 2019)
		Tsetlho (B)	Whole plant	Poultice	Topical	Retained placenta, wounds, dystocia	(Moichwanetse et al, 2020)
		Tsetlho (B)	Whole plant	Unspecified	Unspecified	Retained placenta, bloat	(Van der Merwe et al., 2001)
Triumfetta sonderi Ficalho & Hiern	Malvaceae	Mokuku (B)	Root-bark	Unspecified	Unspecified	Retained placenta	(Van der Merwe et al., 2001)
Tulbaghia acutiloba Harv.	Amaryllidaceae	isihihi (X)	Unspecified	Unspecified	Unspecified	Babesiosis, anaplasmosis	(Masika et al., 1997)
Tulbaghia alliacea L.f.	Amaryllidaceae	isivumba mpunzi (X)	Leaves	Infusion	Unspecified	Anaplasmosis	(Masika & Afolayan, 2003)
Turraea obtusifolia Hochst.	Meliaceae	Mbhovane (V)	Leaves	Grounding	Topical	Wounds	(Luseba & Tshisikhawe, 2013).
Typha capensis (Rohrb.) N.E.Br.	Typhaceae	Unspecified	Unspecified	Decoction	Oral / Topical	Retained placenta	(Roberts 1990)
		Umkhazi (X)	Unspecified	Infusion	Unspecified	Retained placenta	(Beinart & Brown, 2013)

Plants scientific name	Plant family	#Local name	Part used	Preparation method	Administration mode	Diseases	References
Vachellia karroo (Hayne) Banfi & Glasso (Syn: *Acacia karroo* Hayne)	Leguminosae (Fabaceae)	Umnga (X)	Leaves	Maceration	Topical	Maggots, wounds	(Soyelu& Masika, 2009)
		Mooka (B)	Bulb	Maceration	Oral	Retained placenta, Bacterial infection	(Moichwanetse et al, 2020)
		Mooka (B)	Bark	Unspecified	Unspecified	Fracture	(Van der Merwe et al., 2001)
		Mookana (B)	Thorns	Unspecified	Unspecified	Fracture	(Ndou, 2018)
Vachellia nilotica (L.) P.J.H. Hurter & Mabb. (Syn: *Acacia nilotica* (L.) Delile)	Leguminosae (Fabaceae)	Motsha (S)	Unspecified	Unspecified	Topical	Fracture	(Beinart & Brown, 2013)
Vachellia tortilis (Forssk.) Gallaso & Banfi (Syn: *Acacia tortilis* (Forssk.) Hayne)	Leguminosae (Fabaceae)	Mosu (B)	Branch tips	Unspecified	Unspecified	Diarrhoea	(Van der Merwe et al., 2001)
Vepris lanceolata G. Don	Rutaceae	Umzane (X)	Leaves	Infusion	Unspecified	Anaplasmosis	(Beinart & Brown, 2013)
Vitex zeyheri Sond. ex Schauer	Lamiaceae	Mokwele (B)	Leaves	Unspecified	Unspecified	Eye infections	(Van der Merwe et al., 2001)
Volkameria glabra (E.Mey.) Mabb. & Y.W.Yuan (Syn: *Clerodendrum glabrum* E.Mey.)	Lamiaceae	Unspecified	Leaves	Unspecified	Unspecified	Helminths	(Kambizi, 2014)
		Unspecified	Unspecified	Unspecified	Unspecified	Constipation	(Hutchings et al. 1996)
		Unspecified	Leaves	Unspecified	Unspecified	Helminths	(Roberts 1990)
		Uqangazana (X)	Leaves	Infusion	Unspecified	Babesiosis, sores, tick damage	(Beinart & Brown, 2013)
		Mohlokohloko (P)	Latex	Unspecified	Topical	Ticks	(Matlebyane et al., 2010).
		Mohlokohloko (P)	Leaves	Grounding	Topical	Unspecified	(Mogale, 2017).
Waltheria indica L.	Malvaceae	Unspecified	Leaves	Decoction	Oral	Diarrhoea	(Semenya et al., 2019)
Withania somnifera (L.) Dunal	Solanaceae	Ibuvimba (P)	Roots	Unspecified	Unspecified	Anaplasmosis	(Hutchings, 1996)
		Mosalashopeng (B)	Unspecified	Unspecified	Unspecified	Anthrax	(Beinart & Brown, 2013)
		Modikasope (B)	Tubers	Infusion/ Decoction	Oral	Internal sores, pains	(Ndou, 2018)
		Mokukwane (B)	Roots	Unspecified	Unspecified	Diarrhoea	(Van der Merwe et al., 2001)

Plants scientific name	Plant family	#Local name	Part used	Preparation method	Administration mode	Diseases	References
Xanthocercis zambesiaca (Baker) Dumaz-le-Grand	Leguminosae	Mushato (V)	Bark/leaves	Infusion/ Decoction	Oral/ Topical	Diarrhoea	(Luseba & Tshisikhawe, 2013).
		Mushato (V)	Bark	Grounding	Topical	Wounds	(Magwede et al., 2014)
Ximenia americana L.	Olacaceae	Muthanzwa (V)	Rootbark	Grounding	Topical	Wounds	(Chitura et al., 2018)
		Muthanzwa (V)	Leaves, branches	Decoction	Oral	Wounds	(Luseba & Tshisikhawe, 2013).
		Seretologa (B)	Roots	Unspecified	Unspecified	Helminths	(Van der Merwe et al., 2001)
Ximenia caffra Sond.	Olacaceae	Unspecified	Leaves	Infusion	Topical	Dermatophilos is	(Semenya et al., 2019)
Xysmalobium undulatum (L.) W.T.Aiton	Apocynaceae	Leshokgwa/ Poho tshehla (S)	Bulb	Decoction	Unspecified	Anaplasmosis	(Beinart & Brown, 2013)
Zantedeschia aethiopica (L.) Spreng.	Araceae	Inyibiba (X)	Rhizomes	Decoction	Unspecified	Babesiosis	(Masika & Afolayan, 2003)
Zantedeschia albomaculata (Hook.) Baill.	Araceae	Mohaladitwe (S)	Unspecified	Unspecified	Unspecified	Fertility problems, retained placenta	(Beinart & Brown, 2013)
Zanthoxylum capense (Thunb.) Harv.	Rutaceae	Umlung'mabel e (X)	Leaves	Infusion	Unspecified	Anaplasmosis	(Beinart & Brown, 2013)
		uMlungumabel e (X)	Leaves	Infusion	Oral	Anaplasmosis	(Dold & Cocks, 2001)
Ziziphus oxyphylla Edgew. (Syn: *Ziziphus acuminata* Royle)	Rhamnaceae	Sekgalofatshe (B)	Roots	Poultice	Topical	Retained placenta	(Moichwanetse et al, 2020)
Ziziphus mucronata Willd.	Rhamnaceae	Unspecified	Roots	Maceration	Topical	Wounds	(Semenya et al., 2019)
		Mutshetshete (V)	Leaves	Poultice	Topical	Mastitis	(Chitura et al., 2018)
		Mutshetshete (V)	Bark	Infusion	Topical	Wounds	(Magwede et al., 2014)
		Mokgalo (B)	Leaves	Poultice	Topical	Abscess	(Ndou, 2018)
		Sekgalo/Mokg alo (B)	Roots	Poultice	Topical	Retained placenta	(Moichwanetse et al, 2020)

Plants scientific name	Plant family	#Local name	Part used	Preparation method	Administration mode	Diseases	References
		Mokgalo (B)	Roots, leaves	Unspecified	Unspecified	Fertility problem, sores, burns	(Van der Merwe et al., 2001)
Ziziphus zeyheriana Sond.	Rhamnaceae	Sekgalofatshe/ Mokgalofatshe (B)	Roots	Decoction	Oral	Pains (caused by sores and fracture), Diarrhoea	(Ndou, 2018)
		Sekhalo (B)	Roots	Infusion	Unspecified	Diarrhoea	(Beinart & Brown, 2013)
		Sekgalofatshe (B)	Rootstock	Unspecified	Unspecified	Helminths, Diarrhoea	(Van der Merwe et al., 2001)
