# Supplementary material for: Ethnoveterinary Knowledge and Biological Evaluation of Plants Used for Mitigating Cattle Diseases: A Critical Insight Into the Trends and Patterns in South Africa
Source: Front Vet Sci. 2021 Aug 19;8:710884. doi: 10.3389/fvets.2021.710884 (PMC8417044; doi:10.3389/fvets.2021.710884)
Supplement: Supplementary file 2 [file Table_2.pdf]

Supplementary Table S2: List of plant families used for the treatment of diseases in cattle

	Family	Number of plants
1	Achariaceae	1
2	Agavaceae	2
3	Amaranthaceae	2
4	Amaryllidaceae	10
5	Anacardiaceae	7
6	Apiaceae	5
7	Apocynaceae	11
8	Araceae	2
9	Araliaceae	1
10	Asparagaceae	17
11	Balanophoraceae	1
12	Boraginaceae	1
13	Cactaceae	1
14	Campanulaceae	1
15	Cannabaceae	1
16	Capparaceae	3
17	Caricaceae	1
18	Celastraceae	5
19	Clusiaceae	1
20	Colchicaceae	1
21	Combretaceae	6
22	Compositae	24
23	Convolvulaceae	2
24	Cucurbitaceae	2
25	Curtisiaceae	1
26	Dioscoreaceae	2
27	Dryopteridaceae	1
28	Ebenaceae	3
29	Euphorbiaceae	11
30	Fagaceae	1
31	Geraniaceae	5
32	Gesneriaceae	1
33	Gunneraceae	1
34	Hydnoraceae	1
35	Hypoxidaceae	3
36	Icacinaceae	1
37	Iridaceae	2
38	Lamiaceae	13
39	Lauraceae	1
40	Leguminosae	38
41	Loganiaceae	2
42	Loranthaceae	1
43	Malpighiaceae	1
44	Malvaceae	9

	Family	Number of plants
45	Meliaceae	2
46	Melianthaceae	1
47	Menispermaceae	1
48	Moraceae	2
49	Myrtaceae	1
50	Ochnaceae	1
51	Olacaceae	2
52	Oleaceae	1
53	Pedaliaceae	2
54	Peraceae	1
55	Phyllanthaceae	2
56	Phytolaccaceae	2
57	Piperaceae	1
58	Pittosporaceae	1
59	Plumbaginaceae	2
60	Podocarpaceae	1
61	Polygonaceae	2
62	Primulaceae	1
63	Proteaceae	2
64	Rhamnaceae	4
65	Rosaceae	1
66	Rubiaceae	10
67	Rutaceae	4
68	Salicaceae	2
69	Salvadoraceae	1
70	Santalaceae	2
71	Sapindaceae	3
72	Sapotaceae	1
73	Solanaceae	13
74	Thymelaeaceae	1
75	Typhaceae	1
76	Urticaceae	1
77	Verbenaceae	3
78	Vitaceae	7
79	Xanthorrhoeaceae	16
80	Zamiaceae	1
81	Zygophyllaceae	2
