# Supplementary material for: Ethnoveterinary Knowledge and Biological Evaluation of Plants Used for Mitigating Cattle Diseases: A Critical Insight Into the Trends and Patterns in South Africa
Source: Front Vet Sci. 2021 Aug 19;8:710884. doi: 10.3389/fvets.2021.710884 (PMC8417044; doi:10.3389/fvets.2021.710884)
Supplement: Supplementary file 3 [file Table_3.pdf]

Supplementary Table S3: Summary of the medicinal plants used for treating various ailments and diseases in cattle

Category of diseases	Diseases	Common plants	No. of mentioned
**Gastrointestinal problems**			
	Colic	*CyphoStemma natalitium* (Szyszyl.) J.J.M.van der Merwe	1
	Constipation	*Albuca aurea* Jacq.	1
		Allium cepa L.	1
		Aloe marlothii A.Berger	1
		Aloe ferox Mill.	1
		Aloe spicata L.f.	1
		Apodytes dimidiata E.Mey. ex Arn.	1
		Asparagus africanus Lam.	1
		Asparagus falcatus L.	1
		Combretum apiculatum Sond.	1
		Copaiba mopane (J. Kirk ex Benth.) Kuntze	1
		Drimia sanguinea (Schinz) Jessop	1
		Gomphocarpus fruticosus (L.) W.T.Aiton	1
		Helichrysum caespititium (DC.) Sond. ex Harv.	1
		Jatropha curcas L.	1
		Monsonia emarginata L'Hér.	1
		Olea europaea L.	1
		Pterocarpus angolensis DC.	1
		Rhynchosia komatiensis Harms	1
		Ricinus communis L.	1
		Sarcophyte sanguinea Sparrm.	1
		Senna italica Mill.	1
		Solanum panduriforme E.Mey.	1
		Stangeria eriopus (Kunze) Baill.	1
		Volkameria glabra (E.Mey.) Mabb. & Y.W.Yuan	1
		Ximenia caffra Sond.	1
	Diarrhoea	*Aloe marlothii* A.Berger	2
		Aloe greatheadii Schönland	1
		Aloe maculata All.	1

Category of diseases	Diseases	Common plants	No. of mentioned
		Aloe vera (L.) Burm.f.	1
		Balanites maughamii Sprague	2
		Bauhinia thonningii Schum.	1
		Calpurnia aurea (Aiton) Benth.	1
		Capparis tomentosa Lam.	3
		Cassine transvaalensis (Burtt Davy) Codd	1
		Combretum vendae A.E.van Wyk	1
		Dicoma galpinii F.C.Wilson	1
		Dombeya rotundifolia (Hochst.) Planch.	2
		Elephantorrhiza burkei Benth.	1
		Elephantorrhiza elephantina (Burch.) Skeels	3
		Elephantorrhiza obliqua Burtt Davy	1
		Grewia flava DC.	1
		Gymnanthemum coloratum (Willd.) H.Rob. & B.Kahn	1
		Gymnosporia heterophylla (Eckl. & Zeyh.) Loes.	1
		Gymnosporia senegalensis (Lam.) Loes	3
		Harpagophytum procumbens (Burch.) DC. ex Meisn.	1
		Helichrysum caespititium (DC.) Sond. ex Harv.	1
		Hippobromus pauciflorus Radlk.	1
		Hypoxis hemerocallidea Fisch., C.A.Mey. & Avé-Lall.	1
		Indigofera cryptantha Harv.	1
		Indigofera sessilifolia DC.	1
		Jatropha zeyheri Sond.	1
		Osyris lanceolata Hochst. & Steud.	1
		Pelargonium reniforme Curtis	1
		Peltophorum africanum Sond.	2
		Philenoptera violacea (Klotzsch) Schrire	1
		Plectranthus laxiflorus Benth.	1

Category of diseases	Diseases	Common plants	No. of mentioned
		Podocarpus latifolius (Thunb.) R.Br. ex Mirb.	1
		Protea caffra Meisn	2
		Protea welwitschii Engl.	1
		Rhoicissus tridentata (L.f.) Wild & R.B.Drumm.	1
		Schizocarphus nervosus (Burch.) van der Merwe	1
		Sclerocarya birrea (A.Rich.) Hochst.	2
		Searsia pyroides (Burch.) Moffett	1
		Senna sophera (L.) Roxb.	1
		Senna italica Mill.	3
		Solanum incanum L.	1
		Solanum panduriforme E.Mey.	1
		Strychnos henningsii Gilg	1
		Terminalia sericea Burch. ex DC.	1
		Vachellia karroo (Hayne) Banfi & Glasso	1
		Vachellia nilotica (L.) P.J.H. Hurter & Mabb.	1
		Volkameria glabra (E.Mey.) Mabb. & Y.W.Yuan	1
		Withania somnifera (L.) Dunal	2
		Ziziphus mucronata Willd	1
		Ziziphus zeyheriana Sond.	2
	Dysentery	*Clausena anisata* (Willd.) Hook.f. ex Benth.	1
		Elephantorrhiza elephantina (Burch.) Skeels	1
		Protea caffra Meisn.	1
	Gala/gall	*Aloe vera* (L.) Burm.f.	1
		Dicoma galpinii F.C.Wilson	1
		Aloe marlothii A.Berger	1
		Clutia pulchella L.	1
		Philenoptera violacea (Klotzsch) Schrire	1
	Gut condition	*Combretum vendae* A.E.van Wyk	1
	Internal bleeding	*Opuntia ficus-indica* (L.) Mill.	1

Category of diseases	Diseases	Common plants	No. of mentioned
	Internal sore	*Solanum lichtensteinii* Willd.	1
		Withania somnifera (L.) Dunal	1
		Bulbine abyssinica A.Rich.	1
	Intestinal diseases	*Breonadia salicina* (Vahl) Hepper & J.R.I.Wood	1
		Drimia sanguinea (Schinz) Jessop	1
		Senna italica Mill.	1
	Lung sickness	*Erythrophleum lasianthum* Corbishley	1
		Merwilla plumbea (Lindl.) Speta	1
		Phytolacca heptandra Retz.	2
	Stomach pain	*Dicoma galpinii* F.C.Wilson	1
**General system infection**			
	Blood clots	*Peltophorum africanum* Sond.	1
	Bloat	*Gymnosporia senegalensis* (Lam.) Loes	1
		Pouzolzia mixta Solms	1
		Tribulus terrestris L.	1
	Bovine ephemeral fever	*Ptaeroxylon obliquum* (Thunb.) Radlk.	1
		Rhoicissus tomentosa (Lam.) Wild & R.B.Drumm.	2
	Black quarter	*Agapanthus praecox* Willd.	1
		Dicerocaryum eriocarpum (Decne.) Abels	2
		Drimia sanguinea (Schinz) Jessop	1
		Elephantorrhiza elephantina (Burch.) Skeels	2
		Euphorbia cupularis Boiss.	3
		Euphorbia umbellata (Pax) Bruyns	1
		Euphorbia cooperi N.E.Br. ex A.Berger	1
		Grewia flava DC.	1
		Gymnosporia senegalensis (Lam.) Loes	1
		Harpagophytum procumbens (Burch.) DC. ex Meisn.	1
		Helichrysum caespititium (DC.) Sond. ex Harv.	1

Category of diseases	Diseases	Common plants	No. of mentioned
		Olea europaea L.	1
		Ptaeroxylon obliquum (Thunb.) Radlk.	1
		Salvia runcinata L.f.	1
		Schotia brachypetala Sond.	1
		Senecio oxyriifolius DC.	1
		Solanum supinum Dunal	1
		Tephroseris palustris (L.) Rchb.	1
		Tephrosia kraussiana Meissner	1
	Bacterial infection	*Vachellia karroo* (Hayne) Banfi & Glasso	1
	Anthrax	*Aloe spicata* L.f.	1
		Drimia sanguinea (Schinz) Jessop	1
		Gardenia volkensii K.Schum.	1
		Prunus persica (L.) Batsch	1
		Senecio oxyriifolius DC.	1
		Senna italica Mill.	2
		Withania somnifera (L.) Dunal	1
	Anaemia	*Drimia sanguinea* (Schinz) Jessop	1
		Hypoxis hemerocallidea Fisch., C.A.Mey. & Avé-Lall.	1
	Foot and mouth diseases	*Schotia brachypetala* Sond.	1
	Pododermatitis	*Clausena anisata* (Willd.) Hook.f. ex Benth.	1
		Helichrysum appendiculatum (L.f.) Less.	1
		Rauvolfia caffra Sond.	1
	Heart problem	*Hypoxis hemerocallidea* Fisch., C.A.Mey. & Avé-Lall.	1
	Nasal schistosomiasis	*Bridelia micrantha* (Hochst.) Baill.	1
	Pain	*Cadaba aphylla* (Thunb.) Wild	1
		Clutia pulchella L.	1
		Dicoma galpinii F.C.Wilson	1
		Gomphocarpus fruticosus (L.) W.T.Aiton	1

Category of diseases	Diseases	Common plants	No. of mentioned
		Helichrysum caespititium (DC.) Sond. ex Harv.	1
		Hyperacanthus amoenus (Sims) Bridson	1
		Opuntia ficus-indica (L.) Mill.	1
		Senna italica Mill.	1
		Solanum anguivi Lam.	1
		Tapinanthus oleifolius (J.C.Wendl.) Danser	1
		Withania somnifera (L.) Dunal	1
		Ziziphus mucronata Willd.	1
	Paratyphoid	*Acokanthera oppositifolia* (Lam.) Codd	1
		Clutia pulchella L.	1
		Dalbergia obovata E.Mey	1
		Spirostachys africana Sond.	1
		Strychnos henningsii Gilg	1
	Shivering endlessly	*Cyphia stramonium* N.E. Br.	1
	Shock	*Rhoicissus tridentata* (L.f.) Wild & R.B.Drumm.	1
	Sores	*Aloe spicata* L.f.	1
		Bulbine abyssinica A.Rich.	1
		Dioscorea dregeana (Kunth) T.Durand & Schinz	1
		Drimia sanguinea (Schinz) Jessop	2
		Hypoxis hemerocallidea Fisch., C.A.Mey. & Avé-Lall.	1
		Solanum anguivi Lam.	1
		Solanum incanum L.	1
		Solanum lichtensteinii Willd.	1
		Volkameria glabra (E.Mey.) Mabb. & Y.W.Yuan	1
		Withania somnifera (L.) Dunal	1
		Ziziphus mucronata Willd.	1
	Sweating sickness	*Osyris lanceolata* Hochst. & Steud.	1
		Spirostachys africana Sond.	1

Category of diseases	Diseases	Common plants	No. of mentioned
**Fertility/ Reproduction disorders**			
	Abortion	*Boophone* disticha (L.f.) Herb.	2
		Hypoxis hemerocallidea Fisch., C.A.Mey. & Avé-Lall.	1
		Rhoicissus tridentata (L.f.) Wild & R.B.Drumm.	1
	Dystocia	*Aloe marlothii* A.Berger	1
		Azima tetracantha Lam.	1
		Carissa bispinosa (L.) Desf. ex Brenan	1
		Cassytha filiformis L.	1
		Combretum vendae A.E.van Wyk	1
		Dicerocaryum eriocarpum (Decne.) Abels	1
		Dicerocaryum senecioides (Klotzsch) Abels	1
		Dichrostachys cinerea (L.) Wight & Arn.	1
		Podocarpus latifolius (Thunb.) R.Br. ex Mirb.	1
		Solanum lichtensteinii Willd.	1
		Tribulus terrestris L.	1
	Endometritis	*Cussonia spicata* Thunb.	1
		Exomis microphylla (Thunb.) Aellen	1
		Olea europaea L.	1
	Fertility problems	*Boophone disticha* (L.f.) Herb.	1
		Combretum paniculatum Vent.	1
		Croton gratissimus Burch.	1
		Englerophytum magalismontanum (Sond.) T.D.Penn.	1
		Eucomis bicolor Baker	1
		Grewia flava DC.	1
		Hypoxis hemerocallidea Fisch., C.A.Mey. & Avé-Lall.	1
		Zantedeschia aethiopica (L.) Spreng.	1
		Teucrium trifidum Retz.	1
		Trema orientalis (L.) Blume	1

**Category of diseases**	**Diseases**	**Common plants**	**No. of mentioned**
	Hastens oestrus	*Acacia decurrens* Willd.	1
		Acacia mearnsii De Wild.	1
		Aloe vera (L.) Burm.f.	1
		Ziziphus zeyheriana Sond.	1
	Retained placenta	*Aloe marlothii* A.Berger	1
		Aloe greatheadii Schönland	1
		Aloe tenuior Haw	1
		Aloe vera (L.) Burm.f.	1
		Aloe zebrina Baker	1
		Asparagus setaceus (Kunth) Jessop	1
		Bolusanthus speciosus (Bolus) Harms	1
		Boophone disticha (L.f.) Herb.	2
		Breonadia salicina (Vahl) Hepper & J.R.I.Wood	1
		Cassia abbreviata Oliv.	1
		Centella asiatica (L.) Urb.	1
		Chlorophytum cremnophilum Van Jaarsv.	1
		Cissus quadrangularis L.	1
		Cussonia spicata Thunb.	2
		Dicerocaryum eriocarpum (Decne.) Abels	3
		Dicerocaryum senecioides (Klotzsch) Abels	2
		Dichrostachys cinerea (L.) Wight & Arn.	1
		Drimia altissima (L.f.) Ker Gawl.	1
		Drimia sanguinea (Schinz) Jessop	3
		Dryopteris athamantica (Kunze) Kuntze	1
		Elephantorrhiza elephantina (Burch.) Skeels	3
		Erythrina caffra Thunb.	1
		Euphorbia sp.	1
		Ficus sur Forssk.	3

Category of diseases	Diseases	Common plants	No. of mentioned
		Gomphocarpus fruticosus (L.) W.T.Aiton	2
		Grewia lasiocarpa E.Mey. ex Harv	1
		Gunnera perpensa L.	2
		Harpagophytum procumbens (Burch.) DC. ex Meisn.	1
		Hibiscus malacospermus (Turcz.) E.Mey. Ex Harv.	1
		Hibiscus diversifolius Jacq.	1
		Hypoxis hemerocallidea Fisch., C.A.Mey. & Avé-Lall.	1
		Jatropha zeyheri Sond.	2
		Kiggelaria africana L.	1
		Melia azedarach L.	1
		Olea europaea L.	1
		Opuntia ficus-indica (L.) Mill.	1
		Peltophorum africanum Sond.	2
		Pentanisia prunelloides (Klotzsch) Walp.	1
		Plectranthus laxiflorus Benth.	1
		Podocarpus latifolius (Thunb.) R.Br. ex Mirb.	1
		Pouzolzia mixta Solms	2
		Rhoicissus digitata (L. f.) Gilg & M. Brandt	1
		Rumex lanceolatus Thunb.	1
		Salix capensis Thunb.	2
		Searsia lancea (L.f.) F.A.Barkley	1
		Senna sophera (L.) Roxb.	1
		Senna italica Mill.	1
		Senna petersiana (Bolle) Lock	1
		Solanum aculeastrum Dunal	1
		Solanum supinum Dunal	1
		Tapinanthus oleifolius (J.C.Wendl.) Danser	1
		Tephrosia macropoda (E.Mey.) Harv.	1

Category of diseases	Diseases	Common plants	No. of mentioned
		Tribulus terrestris L.	3
		Turraea obtusifolia Hochst.	1
		Typha capensis (Rohrb.) N.E.Br.	1
		Vachellia karroo (Hayne) Banfi & Glasso	1
		Zantedeschia aethiopica (L.) Spreng.	1
		Zanthoxylum capense (Thunb.) Harv.	1
		Ziziphus mucronata Willd.	1
	Umbilical cord inflammation	*Asparagus laricinus* Burch.	1
		Asparagus suaveolens Burch.	1
	Urinary problem	*CyphoStemma cirrhosum* (Thunb.) Desc. ex Wild & R.B.Drumm	1
	Uterine infections	*Asparagus laricinus* Burch.	1
		Asparagus suaveolens Burch.	1
	Vaginal discharge	*Pouzolzia mixta* Solms	1
	Vaginitis	*Cussonia spicata* Thunb.	1
		Exomis microphylla (Thunb.) Aellen	1
		Olea europaea L.	1
	Venereal bulls	*Boophone disticha* (L.f.) Herb.	1
**Skin problem**			
	Burns	*Aloe greatheadii* Schönland	1
		Aloe zebrina Baker	1
		Ziziphus mucronata Willd.	1
	Abscess	*Acrotome inflata* Benth.	1
		Aloe zebrina Baker	1
		Ziziphus mucronata Willd.	1
	Dermatophytosis	*Solanum hermannii* Dunal	1
		Solanum campylacanthum Hochst.	1
		Solanum mauritianum Scop.	1
	Lumpy skin diseases	*Cissus quadrangularis* L.	1
		Searsia lancea (L.f.) F.A.Barkley	1

Category of diseases	Diseases	Common plants	No. of mentioned
	Mange	*Elephantorrhiza elephantina* (Burch.) Skeels	1
	Myiasis	*Protorhus longifolia* (Bernh.) Engl.	1
	Warts	*Solanum campylacanthum* Hochst.	1
	Wounds	*Acanthospermum hispidum* DC.	1
		Aloe ferox Mill.	1
		Aloe vera (L.) Burm.f.	1
		Aloe zebrina Baker	3
		Amaranthus blitum L.	1
		Boophone disticha (L.f.) Herb.	1
		Calpurnia aurea (Aiton) Benth.	1
		Cassia abbreviata Oliv.	1
		Cassytha filiformis L.	1
		Cissampelos capensis L.f.	1
		Cissus quadrangularis L.	1
		Cyclospermum leptophyllum (Pers.) Sprague	1
		Datura stramonium L.	1
		Dioscorea dregeana (Kunth) T.Durand & Schinz	1
		Diospyros lycioides Desf.	1
		Dodonaea viscosa (L.) Jacq.	1
		Drimia sanguinea (Schinz) Jessop	1
		Elephantorrhiza elephantina (Burch.) Skeels	1
		Erythrina lysiStemon Hutch.	1
		Euphorbia cupularis Boiss.	1
		Ficus sur Forssk.	1
		Ficus thonningii Blume	1
		Gomphocarpus fruticosus (L.) W.T.Aiton	1
		Grewia occidentalis L.	1
		Gymnanthemum coloratum (Willd.) H.Rob. & B.Kahn	1

Category of diseases	Diseases	Common plants	No. of mentioned
		Haplocarpha scaposa Harv.	1
		Harpephyllum caffrum Bernh.	1
		Helichrysum kraussii Sch.Bip.	1
		Hippobromus pauciflorus Radlk.	1
		Jatropha curcas L.	1
		Lippia javanica (Burm.f.) Spreng.	1
		Monsonia emarginata L'Hér	1
		Nicotiana tabacum L.	1
		Peltophorum africanum Sond.	1
		Pentanisia prunelloides (Klotzsch) Walp.	1
		Persea americana Mill.	1
		Phyllanthus parvulus Sond.	1
		Phytolacca octandra L.	1
		Pittosporum viridiflorum Sims	2
		Protorhus longifolia (Bernh.) Engl.	1
		Prunus persica (L.) Batsch	3
		Ptaeroxylon obliquum (Thunb.) Radlk.	1
		Pterocarpus angolensis DC.	1
		Rapanea melanophloeos (L.) Mez	1
		Ricinus communis L.	1
		Schotia brachypetala Sond.	1
		Senecio tamoides DC.	1
		Sideroxylon inerme L.	1
		Solanum aculeastrum Dunal	1
		Solanum panduriforme E. Mey.	1
		Spirostachys africana Sond.	1
		Tagetes minuta L.	1
		Terminalia sericea Burch. ex DC.	1

Category of diseases	Diseases	Common plants	No. of mentioned
		Tribulus terrestris L.	1
		Tulbaghia alliacea L.f.	1
		Xanthocercis zambesiaca (Baker) Dumaz-le-Grand	2
		Ximenia americana L.	2
		Ziziphus oxyphylla Edgew.	1
		Ziziphus mucronata Willd.	1
**Internal and external parasites**			
	Flea eradication	*Dicerocaryum senecioides* (Klotzsch) Abels	1
	Helminths	*Albuca aurea* Jacq.	2
		Aloe ferox Mill.	2
		Aloe greatheadii Schönland	1
		Aloe spicata L.f.	1
		Aloe zebrina Baker	1
		Apodytes dimidiata E.Mey. ex Arn	1
		Asparagus virgatus Baker	1
		Brachylaena discolor DC.	1
		Bulbine latifolia (L.f.) Spreng.	1
		Cassia abbreviata Oliv.	3
		Cassine aethiopica Thunb.	1
		Cassine transvaalensis (Burtt Davy) Codd	1
		Clausena anisata (Willd.) Hook.f. ex Benth.	1
		Combretum vendae A.E.van Wyk	1
		Dicerocaryum eriocarpum (Decne.) Abels	1
		Drimia sanguinea (Schinz) Jessop	3
		Elephantorrhiza elephantina (Burch.) Skeels	2
		Gardenia jasminoides J.Ellis	1
		Gymnanthemum coloratum (Willd.) H.Rob. & B.Kahn	1
		Gymnanthemum corymbosum (Thunb.) H.Rob	2

Category of diseases	Diseases	Common plants	No. of mentioned
		Helichrysum milliganii Hook.f.	1
		Hibiscus diversifolius Jacq.	1
		Jatropha curcas L.	1
		Leonotis leonurus (L.) R.Br.	1
		Leonotis ocymifolia (Burm.f.) Iwarsson	1
		Ozoroa paniculosa (Sond.) R.Fern. & A.Fern.	1
		Pappea capensis Eckl. & Zeyh.	1
		Pelargonium reniforme Curtis	1
		Peltophorum africanum Sond.	1
		Phytolacca octandra L.	1
		Ptaeroxylon obliquum (Thunb.) Radlk.	1
		Rhoicissus tomentosa (Lam.) Wild & R.B.Drumm.	2
		Rhoicissus tridentata (L.f.) Wild & R.B.Drumm.	1
		Rhynchosia komatiensis Harms	1
		Rumex acetosa L.	1
		Salix mucronata Thunb.	1
		Schkuhria pinnata (Lam.) Kuntze ex Thell.	1
		Seddera suffruticosa Hallier f.	1
		Senna italica Mill.	1
		Stangeria eriopus (Kunze) Baill.	1
		Tarchonanthus camphoratus L	1
		Tephrosia macropoda (E.Mey.) Harv.	1
		Vitex zeyheri Sond. ex Schauer	1
		Volkameria glabra (E.Mey.) Mabb. & Y.W.Yuan	1
		Ximenia americana L.	1
		Ziziphus zeyheriana Sond.	1
	Insect repellent	*Lippia javanica* (Burm.f.) Spreng	1
	Maggots	*Aloe marlothii* A.Berger	1

Category of diseases	Diseases	Common plants	No. of mentioned
		Callilepis laureola DC.	2
		Calpurnia aurea (Aiton) Benth.	1
	Tick damage	*Volkameria glabra* (E.Mey.) Mabb. & Y.W.Yuan	1
	Vermin	*Tephrosia kraussiana* Meissner	1
**Musculoskeletal systems**			
	Arthritis	*Strychnos henningsii* Gilg	1
	Fracture	*Boophone disticha* (L.f.) Herb.	1
		Dichrostachys cinerea (L.) Wight & Arn.	1
		Ehretia rigida (Thunb.) Druce	1
		Euphorbia cupularis Boiss.	1
		Thesium spp	1
		Haemanthus albiflos Jacq.	1
		Kleinia longiflora DC.	1
		Marrubium vulgare L.	1
		Sclerocarya birrea (A.Rich.) Hochst.	1
		Secamone filiformis J.H. Ross	1
		Seddera suffruticosa Hallier f.	1
		Solanum incanum L.	1
		Vachellia karroo (Hayne) Banfi & Glasso	3
	Ill-thrift	*Jatropha zeyheri* Sond.	1
	Stiff joint	*Tagetes minuta* L	1
	Swelling	*Senecio barbertonicus* Klatt	1
**Respiratory problems**			
	Cough	*Artemisia afra* Jacq. ex Willd.	1
		Elephantorrhiza elephantina (Burch.) Skeels	1
		Helichrysum caespititium (DC.) Sond. ex Harv.	1
		Hippobromus pauciflorus Radlk.	1
			1
	Fever	*Terminalia sericea* Burch. ex DC.	1
	Prevent cold	*Tarchonanthus camphoratus* L.	1

**Category of diseases**	**Diseases**	**Common plants**	**No. of mentioned**
	Pneumonia	*Croton gratissimus* Burch.	1
		Elephantorrhiza elephantina (Burch.) Skeels	1
		Plumbago auriculata Lam	1
		Schizocarphus nervosus (Burch.) van der Merwe	1
		Senna italica Mill	1
**Tick- borne**			
	Babesiosis	*Aloe ferox* Mill	3
		Aloe hahnii Gideon F.Sm. & Klopper	1
		Asparagus laricinus Burch.	1
		Asparagus suaveolens Burch.	1
		Boophone disticha (L.f.) Herb.	1
		Bulbine alooides (L.) Willd.	1
		Cassia abbreviata Oliv.	1
		Cissus quadrangularis L	1
		Combretum caffrum (Eckl. & Zeyh.) Kuntze	1
		Combretum microphyllum Klotzsch	1
		Cussonia spicata Thunb.	2
		Diospyros mespiliformis Hochst. ex A.DC.	1
		Drimia elata Jacq.	1
		Drimia sanguinea (Schinz) Jessop	2
		Elephantorrhiza burkei Benth.	1
		Eucomis autumnalis (Mill.) Chitt.	1
		Ficus sur Forssk.	1
		Gasteria croucheri (Hook.f.) Baker	1
		Gymnanthemum mespilifolium (Less.) H.Rob.	1
		Heteromorpha trifoliata (H.L.Wendl.) Eckl. & Zeyh.	2
		Heteromorpha arborescens (Spreng.) Cham. & Schltdl.	2
		Holarrhena pubescens Wall. ex G.Don	1

Category of diseases	Diseases	Common plants	No. of mentioned
		Hypoxis colchicifolia Baker	2
		Jatropha latifolia Pax	1
		Kedrostis africana (L.) Cogn.	1
		Lippia javanica (Burm.f.) Spreng.	1
		Melia azedarach L.	1
		Olea europaea L.	1
		Osyris lanceolata Hochst. & Steud.	1
		Philenoptera violacea (Klotzsch) Schrire	1
		Plectranthus laxiflorus Benth.	2
		Pterocarpus angolensis DC.	2
		Rhoicissus tomentosa (Lam.) Wild & R.B.Drumm	1
		Rhoicissus tridentata (L.f.) Wild & R.B.Drumm.	1
		Rhus incisa L.f.	1
		Salix capensis Thunb.	2
		Schotia brachypetala Sond.	1
		Schotia latifolia Jacq.	3
		Senna italica Mill.	2
		Senna tora (L.) Roxb.	1
		Strychnos decussata (Pappe) Gilg	1
		Synadenium cupulare L.C. Wheeler	1
		Terminalia sericea Burch. ex DC	1
		Teucrium africanum Thunb.	1
		Triumfetta sonderi Ficalho & Hiern	1
		Volkameria glabra (E.Mey.) Mabb. & Y.W.Yuan	1
		Xysmalobium undulatum (L.) W.T.Aiton	1
	Anaplasmosis	*Acokanthera oblongifolia* (Hochst.) Benth. & Hook.f. ex B.D.Jack	1
		Aloe marlothii A.Berger	1

Category of diseases	Diseases	Common plants	No. of mentioned
		Aloe ferox Mill	3
		Aloe spicata L.f.	1
		Baphia racemosa (Hochst.) Baker	1
		Bersama tysoniana Oliv.	2
		Boophone disticha (L.f.) Herb.	1
		Bulbine abyssinica A.Rich.	1
		Bulbine frutescens (L.) Willd.	1
		Capparis sepiaria L.	1
		Clutia pulchella L.	1
		Cussonia spicata Thunb	3
		Dicerocaryum eriocarpum (Decne.) Abels	1
		Dicoma anomala Sond	2
		Dietes bicolor (Steud.) Sweet ex Klatt	1
		Drimia sanguinea (Schinz) Jessop	2
		Ehretia rigida (Thunb.) Druce	1
		Elephantorrhiza elephantina (Burch.) Skeels	2
		Englerophytum magalismontanum (Sond.) T.D.Penn	1
		Eucomis autumnalis (Mill.) Chitt.	2
		Gomphocarpus fruticosus (L.) W.T.Aiton	1
		Grewia occidentalis L.	1
		Gunnera perpensa L.	1
		Haemanthus albiflos Jacq.	1
		Heteromorpha trifoliata (H.L.Wendl.) Eckl. & Zeyh.	2
		Heteromorpha arborescens (Spreng.) Cham. & Schltdl.	1
		Hypoxis colchicifolia Baker	2
		Ipomoea lacunosa L	1
		Kedrostis africana (L.) Cogn.	2
		Ledebouria revoluta (L.f.) Jessop	2

Category of diseases	Diseases	Common plants	No. of mentioned
		Leonotis leonurus (L.) R.Br.	1
		Leonotis ocymifolia (Burm.f.) Iwarsson	1
		Leucas capensis (Benth.) Engl.	1
		Lippia javanica (Burm.f.) Spreng.	1
		Maerua angolensis DC	1
		Maytenus peduncularis Loes. (Syn: *Maytenus eduncularis*)	1
		Melia azedarach L.	1
		Ochna holstii Engl.	1
		Olea europaea L.	2
		Pappea capensis Eckl. & Zeyh	1
		Pavetta revoluta Hochst	1
		Pelargonium luridum (Andrews) Sweet	1
		Pelargonium reniforme Curtis	1
		Pentanisia prunelloides (Klotzsch) Walp.	1
		Piper nigrum L.	1
		Pittosporum viridiflorum Sims	2
		Plectranthus laxiflorus Benth.	2
		Plumbago zeylanica L.	1
		Podocarpus latifolius (Thunb.) R.Br. ex Mirb.	2
		Ptaeroxylon obliquum (Thunb.) Radl	2
		Pterocarpus angolensis DC.	1
		Rauvolfia caffra Sond.	1
		Rothmannia capensis Thunb.	1
		Salix capensis Thunb.	2
		Salix mucronata Thunb.	1
		Sclerocarya birrea (A.Rich.) Hochst	1
		Senna sophera (L.) Roxb.	1
		Senna italica Mill.	5

Category of diseases	Diseases	Common plants	No. of mentioned
		Terminalia sericea Burch. ex DC.	1
		Tetradenia riparia (Hochst.) Codd	1
		Teucrium africanum Thunb.	1
		Teucrium trifidum Retz	2
		Triumfetta sonderi Ficalho & Hiern	1
		Tulbaghia acutiloba Harv.	1
		Vachellia tortilis (Forssk.) Gallaso & Banfi	1
		Waltheria indica L.	1
		Ximenia caffra Sond.	1
		Zantedeschia albomaculata (Hook.) Baill.	1
		Zanthoxylum capense (Thunb.) Harv.	1
	Cowdriosis	*Alepidea amatymbica* Eckl. & Zeyh.	1
		Aloe maculata All.	1
		Asparagus africanus Lam.	1
		Burchellia bubalina (L.f.) Sims	1
		Carissa spinarum L.	1
		Cassine aethiopica Thunb.	1
		Curtisia dentata (Burm.f.) C.A.Sm.	1
		Cussonia spicata Thunb.	1
		Drimia sanguinea (Schinz) Jessop	1
		Elephantorrhiza elephantina (Burch.) Skeels	3
		Euclea undulata Thunb.	1
		Gnidia capitata L.f.	1
		Grewia occidentalis L.	1
		Hibiscus diversifolius Jacq.	1
		Hippobromus pauciflorus Radlk.	1
		Hypoxis hemerocallidea Fisch., C.A.Mey. & Avé-Lall.	1
		Pelargonium reniforme Curtis	1

Category of diseases	Diseases	Common plants	No. of mentioned
		Printzia pyrifolia Less.	1
		Protea welwitschii Engl.	1
		Quercus robur L.	1
		Rhoicissus tomentosa (Lam.) Wild & R.B.Drumm	1
		Rhoicissus tridentata (L.f.) Wild & R.B.Drumm.	1
		Rumex acetosa L.	1
		Schizocarphus nervosus (Burch.) van der Merwe	1
		Senna italica Mill.	1
		Stangeria eriopus (Kunze) Baill.	1
		Strychnos henningsii Gilg	1
**Eye problem**			
	Blindness	*Tagetes minuta* L.	1
	Conjunctivitis	*Aloe greatheadii* Schönland	1
		Aloe zebrina Baker	1
		Combretum caffrum (Eckl. & Zeyh.) Kuntze	2
		Hippobromus pauciflorus Radlk.	1
		Leonotis leonurus (L.) R.Br.	1
		Melia azedarach L.	1
		Monsonia emarginata L'Hér.	1
		Nicotiana tabacum L.	1
		Peltophorum africanum Sond.	1
		Philenoptera violacea (Klotzsch) Schrire	1
		Schizocarphus nervosus (Burch.) van der Merwe	1
		Vepris lanceolata G. Don	1
	Eye infection	*Asparagus nodulosus* (Oberm.) J.-P.Lebrun & Stork	1
		Euphorbia cupularis Boiss.	1
**Mammary gland condition**			
	Cross infection	*Carissa bispinosa* (L.) Desf. ex Brenan	1

Category of diseases	Diseases	Common plants	No. of mentioned
	Mastitis	*Ziziphus mucronata* Willd.	1
		Dioscorea sylvatica Eckl.	1
		Macrotyloma axillare (E.Mey.) Verdc.	1
